# Exploring Verrucosidin Derivatives with Glucose-Uptake-Stimulatory Activity from *Penicillium cellarum* Using MS/MS-Based Molecular Networking

**DOI:** 10.3390/jof8020143

**Published:** 2022-01-30

**Authors:** Junjie Han, Baosong Chen, Rui Zhang, Jinjin Zhang, Huanqin Dai, Tao Wang, Jingzu Sun, Guoliang Zhu, Wei Li, Erwei Li, Xueting Liu, Wenbing Yin, Hongwei Liu

**Affiliations:** 1 State Key Laboratory of Mycology, Institute of Microbiology, Chinese Academy of Sciences, Beijing 100101, China; hanjj@im.ac.cn (J.H.); chenbs@im.ac.cn (B.C.); zjjsmilence93@163.com (J.Z.); daihq@im.ac.cn (H.D.); wangtao@im.ac.cn (T.W.); sunjz@im.ac.cn (J.S.); liw@im.ac.cn (W.L.); yinwb@im.ac.cn (W.Y.); 2 School of Medical Devices, Shenyang Pharmaceutical University, Shenyang 110016, China; Raynaymond@outlook.com; 3 State Key Laboratory of Bioreactor Engineering, East China University of Science and Technology, Shanghai 200237, China; zhuguoliang@ecust.edu.cn (G.Z.); liuxueting@ecust.edu.cn (X.L.); 4 Institutional Center for Shared Technologies and Facilities, Institute of Microbiology, Chinese Academy of Sciences, Beijing 100101, China; liew@im.ac.cn

**Keywords:** verrucosidins, *Penicillium cellarum*, glucose uptake-stimulating activity, molecular networking

## Abstract

Under the guidance of LC-MS/MS-based molecular networking, seven new verrucosidin derivatives, penicicellarusins A-G (**3**–**9**), were isolated together with three known analogues from the fungus *Penicillium cellarum*. The structures of the new compounds were determined by a combination of NMR, mass and electronic circular dichroism spectral data analysis. The absolute configuration of penicyrone A (**10**) was corrected based on X-ray diffraction analyses. Bioactivity screening indicated that compounds **1**, **2**, and **4** showed much stronger promising hypoglycemic activity than the positive drug (rosiglitazone) in the range of 25–100 μM, which represents a potential new class of hypoglycemic agents. Preliminary structure-activity relationship analysis indicates that the formation of epoxy ring on C_6_-C_7_ in the structures is important for the glucose uptake-stimulating activity. The gene cluster for the biosynthesis of **1–12** is identified by sequencing the genome of *P. cellarum* and similarity analysis with the gene cluster of verrucosidins in *P. polonicum*.

## 1. Introduction

Fungi have attracted much attention of chemists and biologists due to their potential in producing bioactive secondary metabolites with diverse chemical skeletons [1,2]. Verrucosidins produced by *Penicillium* strains belong to a family of highly reducing fungal polyketides that are characterized with 2*H*-pyran-2-one and dicyclic fused 3,6-dioxabicyclo[3.1.0]hexane moieties interlinked by a polyene chain [3,4,5,6]. They have been reported to display important bioactivities, such as antitumor [7,8], antivirus [9], antibacterial [3,10], and neurological activities [11]. In order to explore in depth this kind of compounds with unique chemical structure and diverse biological activities, we explored *Penicillium* strains collected in our lab searching for verrucosidin analogues.

Molecular networking analyses include acquisition and similarity comparison of mass spectral fragment data, cluster grouping and visualization [12,13]. More recently, the MS/MS-based molecular networking has been demonstrated to be powerful in dereplicating known natural products from a targeted extract and searching for new analogues with the specific skeleton.Examples included thermoactinoamide A with moderate antiproliferative activity from *Thermoactinomyces vulgaris* DSM 43016 [14], suffranidines A-C with significant neuritogenic activity from *Flueggea suffruticosa* [15], and trilliumoside D with strong cytotoxicity against MOLT-4 cell lines from *Trillium tschonoskii* maxim [16]. To explore new reducing fungal polyketides from fungi, we applied the LC-MS/MS-based molecular networking for new verrucosidins from *Penicillium* strains using deoxyverrucosidin that was deposited in our compound library as the probing agent.

**Figure 1:**
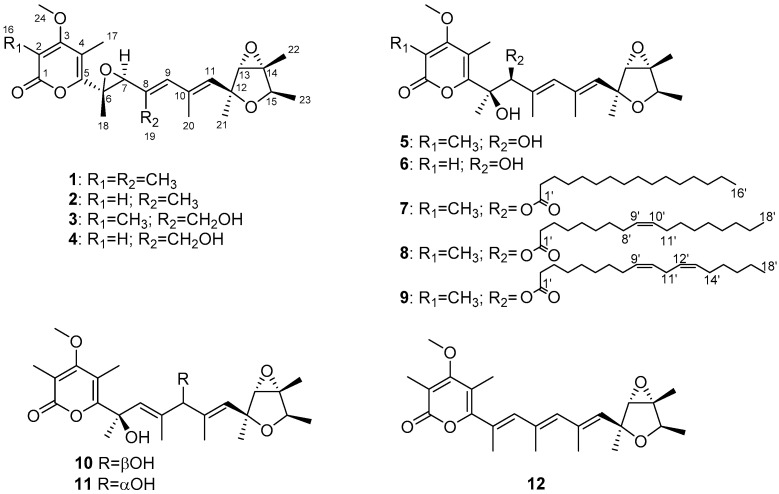
Structures of compounds **1**–**12**.

The EtOAc extracts of *Penicillium* strains fermented on solid culture were first analyzed by high performance liquid chromatography (HPLC) with UV diode array detection (DAD) to find fungi potentially producing verrucosidin derivatives (Figure S1). In this work, the target isolation was further conducted on the selected fungus *P.* *cellarum* YM1 under the guidance of LC-MS/MS-based molecular networking (Figure S2). As a result, seven new verrucosidins, penicicellarusins A-G (**3–9**), as well as five known verrucosidins (compounds **1**, **2** and **10**–**12**) were identified from the culture of *P. cellarum* YM1 (Figure 1). The isolated compounds were evaluated for anti-bacterial effect, cytotoxicity, and glucose uptake-stimulating activities. This work described the details of the isolation, structure elucidation, and biological activities of the isolated secondary metabolites from *P. cellarum* YM1.

## 2. Materials and Methods

### 2.1. General

NMR spectral data were obtained with an AVANCE-500 spectrometer (Bruker, Bremen, Germany) (CDCl_3_*, δ*_H_ 7.26/*δ*_C_ 77.16, and CD_3_OD, *δ*_H_ 3.30/*δ*_C_ 49.9). High-resolution electrospray ionization mass spectrometry (HRESIMS) data and LC-MS/MS measurements were procured on a Q Exactive Orbitrap mass spectrometer (Thermo Fisher Scientific, Waltham, MA, USA) coupled with a LC-30AD series UPLC (Shimadzu, Kyoto, Japan) equipped with an ACQUITY BEH C18 column (Waters, MA, USA; 2.1 × 100 mm, 1.7 μm). UV data, optical rotation, and IR data, were recorded on Genesys-10S UV-Vis spectrophotometer (Thermo Fisher Scientific, Waltham, MA, USA), MCP 200 Automatic Polarimeter (Anton Paar, Graz, Austria) and IS5 FT-IR spectrophotometer (Thermo Fisher Scientific, Waltham, MA, USA) respectively. The CD spectra were measured by a J-815 spectropolarimeter (JASCO, Tsukuba, Japan). Silica gel (Qingdao Haiyang Chemical Co., Ltd., Qingdao, China, 200–300 mesh), Sephadex LH-20 (GE Healthcare, Uppsala, Sweden), and ODS (50 μm, YMC Co., Ltd., Kyoto, Japan) were used for column chromatography. Semi-preparative HPLC was performed on an Agilent 1200 HPLC system equipped with a DAD UV−vis spectrometric detector (Agilent Technologies Inc., CA, USA) using a reversed-phase Eclipse XDB-C8 column (5 μm, 9.4 × 250 mm, Agilent) with a flow rate of 2.0 mL/min and a CHIRALPAK IC column (5 μm, 4.6 × 250 mm, Daicel, Osaka, Japan) with a flow rate of 0.8 mL/min. For gas chromatography-mass spectrometry (GC-MS) a Shimadzu GCMS-QP2010 Ultra system (Shimadzu, Kyoto, Japan) was used. 

### 2.2. Fungal Material

The strain *Penicillium* sp. YM1 used in this work was isolated from mildewed corn, collected in China, in September 2017. The sequences of RPB2 (MT898427), Ben A (MT898428), and CaM (MT898429) of our fungus were deposited in GenBank and employed for phylogenetic analysis. The fungus is similar to *P. cellarum* in forming hyaline, roughened stipes with bearing terminal terverticillate penicillii; and producing typically two rami per stipe, which are usually hyaline, roughened, appressed or only narrowly divergent; and having four to five metulae typically per ramus, which are usually hyaline, roughened, appressed or only narrowly divergent as well; and producing typically six to eight per metula phialides, which are usually hyaline, smooth, ampulliform, slender; and with pale green conidia that were typically smooth, globose to sometimes subglobose [17,18]. The phylogenetic analyses based on a combined dataset of RPB2, Ben A, and CaM was conducted by using PhyML v.3.0, with 1000 bootstrap replicates presented that our taxon grouped with the other taxa of *P. cellarum* with strongly maximum likelihood bootstrap proportions value (Figure S3). In consideration of the morphological features and phylogeny, this fungus was identified as *P. cellarum* YM1.

### 2.3. Fermentation and Extraction

*P. cellarum* was cultured on slant of PDA at 28 °C for 10 days. To prepare inoculum, the spores of the strain on the plate were collected with 0.01% sterile solution of Tween 80 (BTL, Warsaw, Poland) and adjusted to 1 × 10^6^ CFU/mL. A large-scale fermentation was done in 40 × 500 mL Fernbach culture flasks containing 80 g of rice in 110 mL of distilled water (each with 0.5 mL of spore suspension) and incubated at 28 °C for 3 weeks. The fermented rice substrates were extracted with EtOAc (3 × 4 L) with the aid of ultrasonication, and the organic solvent was filtered and evaporated to dryness under vacuum to afford the crude extract (33.7 g).

### 2.4. LC-MS/MS and Molecular Metworking Analysis

LC-MS/MS (MS/MS scan 100−1500 Da) was performed with a Waters ACQUITY BEH C_18_ column (2.1 × 100 mm, 1.7 μm particles) eluted by MeCN−H_2_O (0.005% TFA) (0.01−8 min 5−80% 8−12 min 80−99%, 12−15 min 99%) in a gradient manner. All the MS/MS data files were converted to “.mzML” format files using MSConver software and uploaded on the GNPS Web platform (http://gnps.ucsd.edu (accessed on 6 October 2021)) for MN analysis using Classic mode. For the network creation, a parent mass tolerance of 0.02 Da and a fragment ion tolerance of 0.05 Da were applied. The generated molecular network was visualized in Cytoscape 3.8.2 (www.cytoscape.org (accessed on 6 October 2021)) and guided the isolation of **1–12**. The MS/MS molecular network can be browsed and downloaded on the GNPS Web site with the following link: https://gnps.ucsd.edu/ProteoSAFe/status.jsp?task=8716192add914a1fb3bd8f469f7d2d81 (accessed on 6 October 2021).

### 2.5. Isolation and Characterization Data

The EtOAc fraction was subjected to a silica gel column chromatography (CC) eluting witH-N-hexane/ether-ethyl acetate (*v*/*v*, 100:0, 100:1, 100:2, 100:4, 100:10) and dichloromethane/methanol (*v*/*v*, 100:0, 100:1, 100:2, 100:4, 100:8, 100:12, 100:20, 0:100) to give 13 fractions (PC.1–PC.13). Fractions PC.6, PC.8, and PC.12 containing secondary metabolites with similar UV spectra were selected for further purification.

Fraction PC.6 (1.5 g) was further separated on silica gel column by a gradient elution with methanol-dichloromethane to give 25 fractions (PC.6-1–PC.6-25). PC.6–8 (60 mg) was purified finally by RP-HPLC with acetonitrile-water (63:37) to give **1** (13.5 mg, *t*_R_ 42.3 min) and **2** (6.6 mg, *t*_R_ 31.5 min). Compound **12** (44.5 mg) was obtained from subfractions PC.6–11 (65 mg) by Sephadex LH-20 chromatography eluting with methanol. Compounds **7** (8.6 mg, *t*_R_ 31.1 min), **8** (9.5 mg, *t*_R_ 40.5 min), and **9** (1.6 mg, *t*_R_ 42.5 min) were obtained from PC.6–20 (75 mg) by RP-HPLC using 86% acetonitrile in acidic water (0.005% TFA).

Fraction PC.8 (3.5 g) was separated on an ODS column using a gradient elution with methanol (35%, 55%, 70%, and 100%) in acidic water (0.005%TFA) to afford 15 subfractions (PC.8-1–PC.8–15). Compounds **5** (14.2 mg) and **6** (12.8 mg) were obtained from subfractions PC.8–9 and PC.8–10 by Sephadex LH-20 chromatography eluting with methanol, respectively.

Fraction PC.12 (4.3 g) eluted with CH_2_Cl_2_-acetone (*v*/*v* 20:1) was first separated by ODS using a gradient of increasing methanol (35%, 55%, 70%, and 100%) in water to afford 21 subfractions (PC.12-1–PC.12-21). Compounds **3** (2.5 mg, *t*_R_ 62.5 min) and **4** (3.5 mg, *t*_R_ 30.5 min) were yielded from PC.12-15 (35 mg) by RP-HPLC using 23% acetonitrile in acidic water (0.005% TFA). Subfractions PC.12-6 (60 mg) was purified by RP-HPLC using 27% acetonitrile in water to afford a mixture of **10** and **11** (35.0 mg, *t*_R_ 28.3 min). Enantioseparation of the mixture was carried out on CHIRALPAK IC using isopropanol/*n*-hexane (15:85) as mobile phase to afford **10** (10.0 mg, *t*_R_ 21.5 min) and **11** (11.5 mg, *t*_R_ 23.5 min).

*Penicicellarusin A (**3**).* light yellow oil, [α${]}_{D}^{25}$ + 55.6 (c 0.1 MeOH); UV (MeOH) *λ*_max_ (log *ε*) 240 (3.11), 302 (1.24) nm; IR (neat) *v*_max_ 3429, 2972, 2931, 1703, 1574, 1450, 1378, 1210, 1042, 811 cm^−1^; Positive HRESIMS: *m/z* 455.2041 [M+Na]^+^ (calcd. for C_24_H_32_O_7_Na, 455.2040). ^1^H-NMR and ^13^C-NMR, see Table 1.

*Penicicellarusin B (**4**)*. light yellow oil, [α${]}_{D}^{25}$ + 51.0 (c 0.1 MeOH); UV (MeOH) *λ*_max_ (log *ε*) 241 (3.35), 297 (1.50) nm; IR (neat) *v*_max_ 3420, 2971, 2931, 1700, 1572, 1450, 1377, 1209, 1054, 812 cm^−1^; Positive HRESIMS: *m/z* 441.1890 [M+Na]^+^ (calcd. for C_23_H_30_O_7_Na, 441.1884). ^1^H-NMR and ^13^C-NMR, see Table 1.

*Penicicellarusin C (**5**).* light yellow oil, [α${]}_{D}^{25}$ + 45.0 (c 0.1 MeOH); UV (MeOH) *λ*_max_ (log *ε*) 236 (3.56), 303 (2.57) nm; IR (neat) *v*_max_ 3410, 2974, 2930, 1685, 1560, 1450, 1378, 1224, 1089, 1043, 812 cm^−1^; Positive HRESIMS: *m/z* 443.2047 [M+Na]^+^ (calcd. for C_23_H_32_O_7_Na, 443.2040). ^1^H-NMR and ^13^C-NMR, see Table 2.

*Penicicellarusin D (**6**).* light yellow oil, [α${]}_{D}^{25}$ + 32.9 (c 0.1 MeOH); UV (MeOH) *λ*_max_ (log *ε*) 235 (3.56), 299 (2.54) nm; IR (neat) *v*_max_ 3412, 2974, 2932, 1683, 1559, 1450, 1378, 1225, 1089, 1043, 812 cm^−1^; Positive HRESIMS: *m/z* 457.2190 [M+Na]^+^ (calcd. for C_24_H_34_O_7_Na, 457.2197). ^1^H-NMR and ^13^C-NMR, see Table 2.

*Penicicellarusin E (**7**).* light yellow oil, [α${]}_{D}^{25}$ +7 8.5 (c 0.1 MeOH); UV (MeOH) *λ*_max_ (log *ε*) 235 (3.65), 299 (2.58) nm; IR (neat) *v*_max_ 2975, 2933, 1695, 1555, 1450, 1378, 1224, 1086, 1043, 812 cm^−1^; Positive HRESIMS: *m/z* 695.4490 [M+Na]^+^ (calcd. for C_40_H_64_O_8_Na, 695.4493). ^1^H-NMR and ^13^C-NMR, see Table 3.

*Penicicellarusin F (**8**).* light yellow oil, [α${]}_{D}^{25}$ + 81.0 (c 0.1 MeOH); UV (MeOH) *λ*_max_ (log *ε*) 236 (3.38), 290 (2.54) nm; IR (neat) *v*_max_ 2972, 2932, 1689, 1557, 1450, 1378, 1225, 1088, 1045, 811 cm^−1^; Positive HRESIMS: *m/z* 721.4658 [M+Na]^+^ (calcd. for C_42_H_66_O_8_Na, 721.4650). ^1^H-NMR and ^13^C-NMR, see Table 3.

*Penicicellarusin G (**9**).* light yellow oil, [α${]}_{D}^{25}$ + 76.5 (c 0.1 MeOH); UV (MeOH) *λ*_max_ (log *ε*) 236 (3.38), 290 (2.54) nm; IR (neat) *v*_max_ 2974, 2932, 1720, 1570, 1455, 1378, 1209, 1054, 812 cm^−1^; Positive HRESIMS: *m/z* 719.4500 [M+Na]^+^ (calcd. for C_42_H_64_O_8_Na, 719.4493). ^1^H-NMR and ^13^C-NMR, see Table 3.

### 2.6. X-ray Crystallographic Analysis of Compound **1** and **10**

#### 2.6.1. Penicicellarusin A (**1**)

Colorless needles of compound **1** were obtained from ethyl ether. Data collection was performed on a Eos CCD (Bruker, Bremen, Germany) using graphite-monochromated Cu K_α_ radiation, *λ* = 1.54184 Å at 100.00(10) K. Crystal data: C_24_H_32_O_6_, *M* = 416.49, space group orthorhombic, *P2_1_2_1_2_1_*; unit cell dimensions were determined to be a = 5.70420(10) Å, b = 11.7760(2) Å, c = 33.3228(6) Å, *α* = *β* = *γ* = 90.00°, *V* = 2238.38(7) Å^3^, Z = 4, ρ_calc_ =1.236 mg/mm^3^, *F* (000) = 896.0, *μ* (Cu K_α_) = 0.715 mm^−1^. 16083 unique reflections were collected to 2*θ_max_* = 144.15°, in which 4334 reflections were observed [F^2^ > 4*σ* (F^2^)]. The structure refinements were conducted by a previously reported method [19]. The final refinement gave R_1_ = 0.0357, *w*R_2_ = 0.0852 (*w* = 1/*σ*|F|^2^), and S = 1.047. CCDC 2039557 contains the supplementary crystallographic data for **1**. These data can be obtained from The Cambridge Crystallographic Data Centre via www.ccdc.cam.ac.uk/data_request/cif (accessed on 20 October 2020).

#### 2.6.2. Penicyrone A (**10**)

Colorless needles of compound **10** from methanol were obtained. Data collection was performed on a Eos CCD using graphite-monochromated Cu K_α_ radiation, *λ* = 1.54184 Å at 100.00(10) K. Crystal data: C_24_H_34_O_7_, *M* = 434.519, space group monoclinic, *P2_1_*; unit cell dimensions were determined to be a = 5.96230(10) Å, b = 14.1879(3) Å, c = 14.0680(6) Å, *α* = *γ* = 90.00°, *β* = 95.885° *V* = 1183.78(4) Å^3^, Z = 2, ρ_calc_ =1.219 mg/mm^3^, *F* (000) = 468.0, *μ* (Cu K_α_) = 0.728 mm^−1^. 12357 unique reflections were collected to 2*θ_max_* = 140.124°, in which 4418 reflections were observed [F^2^ > 4*σ* (F^2^)]. The structure refinements were conducted by the same method as described for compound **1**. The final refinement gave R_1_ = 0.0341, *w*R_2_ = 0.0842 (*w* = 1/*σ*|F|^2^), and S = 1.048. CCDC 2039558 contains the supplementary crystallographic data for **10**. These data can be obtained from The Cambridge Crystallographic Data Centre via www.ccdc.cam.ac.uk/data_request/cif (accessed on 20 October 2020).

### 2.7. Alkaline Hydrolysis of Compound ***8*** and ***9***

Alkaline hydrolysis reaction was carried out following a previously described method [20]. Each compound (2.0 mg) was dissolved and hydrolyzed with 2 M NaOH/MeOH at 25 °C for 3 h. Then neutralized with 1 N HCl/MeOH and extracted with chloroform for two times (10 mL × 2). Methyl esters of the fatty acids were identified by GC-MS. The GC-MS was operated in EI mode (70 eV) scanning from 40 to 500 amu.

### 2.8. Bioinformatic Analyses

To identify Biosynthetic Gene Clusters (BGCs) in the genomes of *P. cellarum* YM1, antiSMASH 6.2 was used and only clusters containing a putative PKS similar to both VerA and CtvA protein were further considered [21,22]. The proteins in these clusters were additionally blasted against *P. polonicum* and *Aspergillus terreus* var*. aureus* to verify their presence. To find functional domains and predict a putative function, we resort to NCBI BLAST using Non-Redundant database and Interproscan.

### 2.9. Computation Section

Systematic conformational analyses for **5a**, **5b**, **5c** and **5d** were performed using the CONFLEX softwre (version 7 Rev. A; CONFLEX Corporation, Tokyo, Japan) via the MMFF94 molecular mechanics force field. Using TDDFT at B3LYP/6-31+G(d,p) basis set level, the MMFF94 conformers were further optimized in methanol with PCM model. The stationary points have been checked as the true minima of the potential energy surface by verifying that they do not exhibit vibrational imaginary frequencies. ECD spectra were calculated by TD-DFT using a Gaussian function at the PBE1PBE/6-311G* level. Using Boltzmann statistics, equilibrium populations of conformers at 298.15 K were calculated from their relative free energies (∆G). According to Boltzmann weighting of main conformers, the overall ECD spectra were then generated [23].

### 2.10. Evaluation of Biological Activities

#### 2.10.1. Antimicrobial Bioassay

Assay for antibacterial activities including *Staphylococcus aureus* (ATCC 6538 and CGMCC 1.2465), meticillin-resistant *S. aureus* (MRSA, clinical isolates, Beijing Chao-yang Hospital, Beijing, China), *Enterococcus faecalis* (clinical isolates, Beijing Chao-yang Hospital), *Bacillus subtilis* (ATCC 6633), and antifungal activities including *Candida albicans* (ATCC 18804), and *Aspergilus fumigatus* (CGMCC 3.5835) were carried out as previously described method [24]. The inhibition rate was calculated and plotted versus test concentrations to afford the MIC. MIC values were defined as the minimum concentration of compounds that inhibited visible microbial growth. All the experiments were performed in triplicate.

#### 2.10.2. Cytotoxicity Assay

Cytotoxicity test against A549, HepG2, and K562 cell lines was carried out as previously described method [25]. Taxol, 5-flourouracil, and cisplatin were used as the positive controls.

#### 2.10.3. 2-[N-(7-Nitrobenz-2-oxa-1,3-diazol-4-yl)amino]-2-deoxy-d-glucose (2-NBDG) Glucose Uptake Assay

This experiment was consistent with those reported in our previous work [26]. The HepG2 hepatoma cells were cultured in DMEM supplemented with 10% fetal bovine serum (FBS; Gibco, NY, USA), 100 U/mL penicillin/streptomycin. The cells reaching confluence were treated with 10^−6^ M insulin for 24 h to generate insulin resistance. Compounds or positive drug (rosiglitazone) were mixed and incubated for 24 h, with the final concentration of 100, 50, 25, and 12.5 μM; then, 100 nM of insulin was added and incubated for 30 min at 37 °C followed by addition of 50 μM (2-NBDG). After that, cells were washed with ice-cold PBS and 100 μL FBS-free DMEM was added to each well. The level of 2-NBDG uptake was determined on microplate reader (Bio-Tek Instruments, VT, USA) at 485 nm excitation and 528 nm emission. All data were handled with GraphPad Prism 5 and reported as mean ± SD of three independent experiments.

## 3. Results

In this study, the MS/MS-based molecular networking strategy was applied for target isolation of new verrucosidins. First, *Penicillium* strains were cultured on rice substrates and the resulting EtOAc extracts was screened by HPLC-UV-DAD analysis (Figure S1). Then, the ethyl acetate extract of *P. cellarum* YM1 that produced secondary metabolites with similar retention time and UV characteristics to those of deoxyverrucosidin was further investigated by UPLC-HRMS/MS. The LC-MS/MS data were used to generate a visualized molecular networking that was further annotated by Cytoscape 3.8.2 (Figure S2).

**Figure 2:**
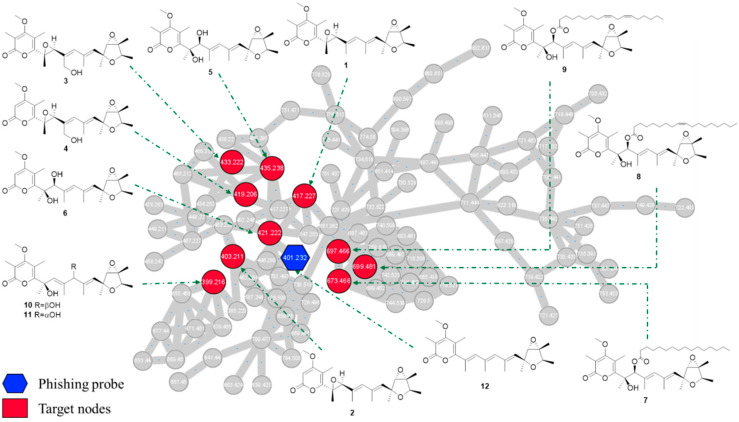
The subnetwork of tandem MS/MS molecular working for crude extracts of the fungus *P. cellarum*. The entire network and subnetwork are presented in Figure S2.

In details, the HPLC-HRMS/MS analysis in the positive ion mode was conducted on the ethyl acetate extract from *P. cellarum* YM1 with deoxyverrucosidin as the phishing probe. The obtained fragmentation data were organized by molecular networking, yielding a metabolite-level view of the data. Individual MS/MS spectrum was organized into 106 clusters consisting of 899 connected nodes (Figure S2). Using the MS/MS data of deoxyverrucosidin as “seed” spectra, an initial focal point (a blue hexagon with *m/z* 401.232) was generated in the global molecular networking. A close examination of the molecular network indicated some nodes connected to deoxyverrucosidin (Figure 2), which predicted the presence of potential natural analogs. Under the guidance of MS/MS-based molecular networkings, seven new verrucosidins, namely penicicellarusins A-I (**3–9**), in addition to five known polyketides verrucosidin (**1**) [4,27], normethylverrucosidin (**2**) [27], penicyrone A-B (**10–11**) [28], and deoxyverrucosidin (**12**) [29] were obtained by the isolation workflow. The structures of known compounds were determined by comparing their NMR and MS data with literature data.

**Figure 3:**
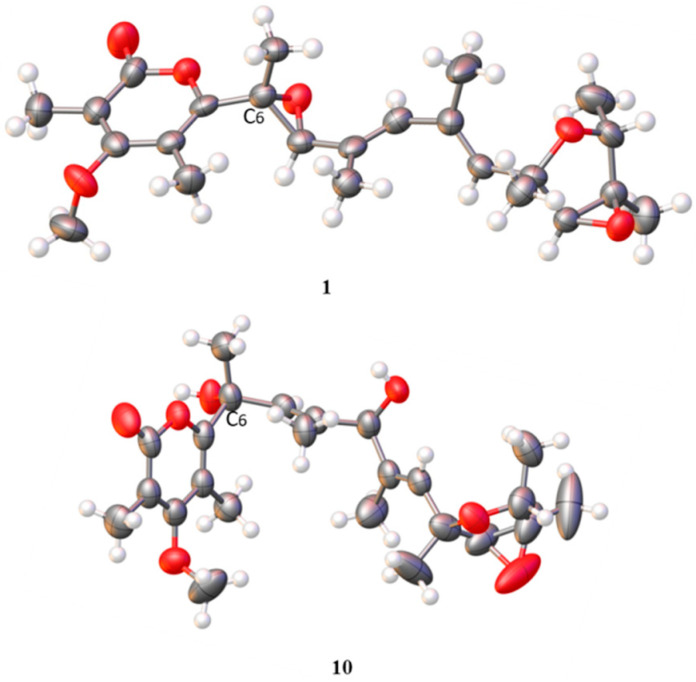
The X-ray crystallographic structure of **1** and **10**.

Compound **1** was obtained as white needle-like crystals and identified as verrucosidin by comparison of the NMR data reported in the literature [4,27] and the single-crystal X-ray crystallographic analysis (Figure 3).

Penicicellarusin A (**3**) was isolated as yellow oil with a molecular formula C_24_H_32_O_7_ (indicating nine degrees of unsaturation) as deduced by HRESIMS data ([M+Na]^+^ *m/z* 455.2041; calcd. 455.2040). The ^1^H-, ^13^C-NMR and HSQC spectra of **3** revealed the presence of eight methyls, including one oxygenated one [*δ*_H_*/δ*_C_ 1.23 (3H, d, 6.8 Hz) /19.2, 1.40 (3H, s)/22.1, 1.44 (3H, s)/15.7, 1.48 (3H, s)/13.8, 1.98 (3H, s)/18.7, 2.04 (3H, s)/10.4, 2.12 (3H, s)/9.7, and 3.91(3H, s)/61.3], one hydroxymethyl [*δ*_H_/*δ*_C_ 4.36 (d, *J* = 12.2 Hz), 4.41 (d, *J* = 12.2 Hz)/59.6], five methines including three oxygenated [*δ*_H_*/δ*_C_ 3.60 (s)/68.7, 3.81 (s)/64.6, 4.09 (d, 6.8 Hz)/78.4] and two *sp*^2^ methines [*δ*_H_*/δ*_C_ 5.60 (brs) /133.9, 5.98 (brs)/134.9]. The NMR data of compound **3** were similar with those of verrucosidin (**1**) [27], indicating the presence of a 3,6-dioxabicyclic[3.1.0]hexane moiety, a 2*H*-pyran-2-one moiety, and a polyene chain in **3** (Figure 1).

**Figure 4:**
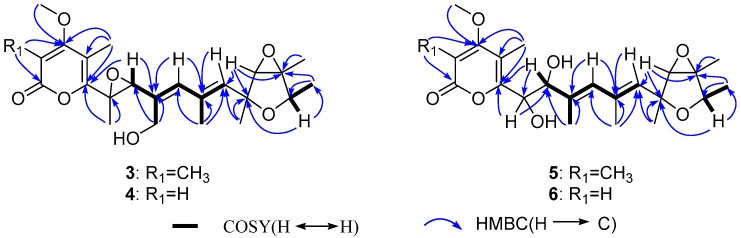
Selected key HMBC and ^1^H-^1^H COSY correlations of **3–6**.

The HMBC correlations were detected from H_3_-21 to C-11 and C-12, from H-13 to C-12 and C-14, from H_3_-22 to C-14, from H_3_-23 to C-14 and C-15, from H-15 to C-12, C-14 and C-23, which together with the ^1^H-^1^H COSY correlations of H-15-H_3_-23 confirmed the presence of the 3,6-dioxabicyclic[3.1.0]hexane moiety. The HMBC correlations of H_3_-16 to C-1, C-2, and C-3, H_3_-24 to C-3, H_3_-17 to C-3, C-4, and C-5, and as well as the chemical shifts of C-1 (*δ* 167.5), C-2 (*δ* 111.6), C-3 (*δ* 170.2), C-4 (*δ* 112.2), and C-5 (*δ* 157.3) completed the assignment of α-pyranone moiety. Furthermore, the ^1^H-^1^H COSY correlations of H-7-H_3_-19-H-9-H_3_-20-H-11 together with the HMBC correlations from H_3_-18 to C-5 and C-6, from H-7 to C-5, C-6, and C-8, from H_3_-19 to C-7 and C-8, from H-9 to C-7, C-8, and C-10, from H_3_-20 to C-9, C-10, and C-11, and from H-11 to C-10 and C-12 supported a heptadiene moiety which was connected with α-pyranone moiety through C-5 and linked with 3,6-dioxabicyclic[3.1.0]hexane moiety through C-12 (Figure 4).

**Figure 5:**
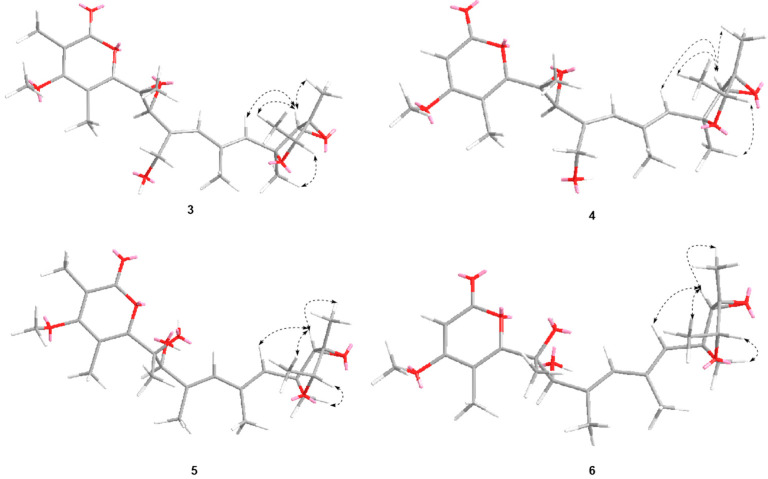
Selected key NOE correlations of **3–6**.

The relative configuration of **3** was confirmed by NOESY experiment (Figure 5). The NOE correlations H-11 (*δ* 5.60) to H_3_-23 (*δ* 1.23) and H-13 (*δ* 3.60), H_3_-22 (*δ* 1.48) to H-13 and H_3_-23, and H-15 (*δ* 4.09) to H_3_-21 (*δ* 1.40), indicated that H_3_-22, H_3_-23, H-11, and H-13 were on the same face, while H_3_-21 and H-15 were on the opposite face. The geometry of C_8_=C_9_ and C_10_=C_11_ were confirmed to be *E* by the NOE correlations of H-7 (*δ* 3.81) with H-9 (*δ* 5.98) and H_3_-20 with H_3_-21.

**Figure 6:**
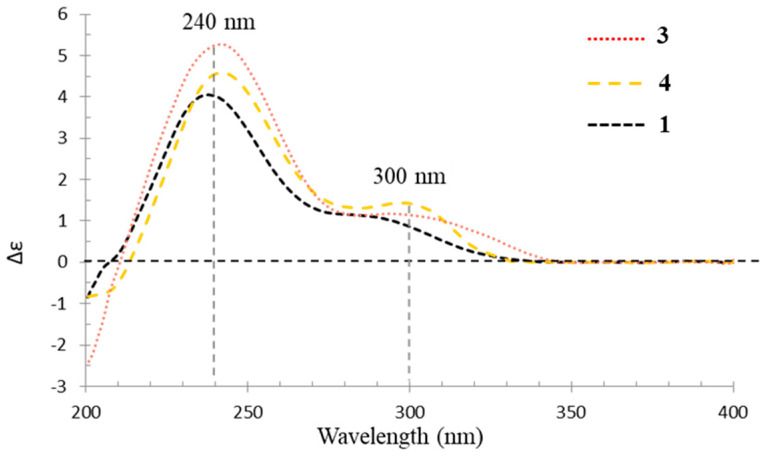
Experimental CD spectra of **1**, **3**, and **4** in MeOH.

In the experimental ECD spectrum, compound **3** showed similar Cotton effects as verrucosidin (**1**) (Figure 6), supporting the same configuration at C-6 and C-7 between **1** and **3**. Thus, compound **3** was assigned a 6*R*, 7*S*, 12*S*, 13*S*, 14*R*, 15*R* configuration and named penicicellarusin A.

Penicicellarusin B (**4**) was obtained as yellow oil with the molecular formula C_23_H_30_O_7_ and nine degrees of unsaturation, as deduced from HRESIMS data. The 1D NMR spectroscopic data of **4** (Table 1) were similar with those of **3**, except for the lack of a singlet methyl group and the presence of one additional olefinic proton at *δ* 5.66 in **4**. HMBC correlations of H-2 (*δ* 5.66) to C-1, C-3 and C-4, H_3_-17 to C-3 and C-5, H_3_-24 to C-3 confirmed the structural changes on the 2*H*-pyran-2-one moiety in **4**. A further comprehensive analysis of its ^1^H-^1^H COSY, HMQC, and HMBC spectra assigned the planar structure of **4** (Figure 4).

The NOESY correlations of H-11 with H-13 and H_3_-22, H_3_-23 with H-13 and H_3_-22, H-15 with H_3_-21, H-7 with H-9 and H_3_-17, H_3_-20 with H_3_-21 supported the same relative configurations for double bonds and 3,6-dioxabicyclic[3.1.0]hexane moiety between 4 and compound **3** (Figure 5). Compound **4** showed similar Cotton effects in the experimental electronic circular dichroism (ECD) spectrum with those of **3** (Figure 6), indicating it has the absolute configuration of 6*R*, 7*S*, 12*S*, 13*S*, 14*R*, and 15*R*, as described in **3**.

Compound **5** was assigned the molecular formula of C_24_H_34_O_7_ (eight degree of unsaturation) on the basis of its HRESIMS at *m/z* 457.2190 [M+Na]^+^ and NMR data (Table 2). The ^1^H-, ^13^C-NMR, and UV spectra of **5** were similar with those of verrucisidinol [6], with the notable difference in the ^1^H-NMR data of C-3, C-4, C-5, and H-7 (Table 2). A comprehensive analysis of its 2D NMR spectra including ^1^H-^1^H COSY, HMQC, and HMBC experiments confirmed the planar structure of **5** (Figure 4).

**Figure 7:**
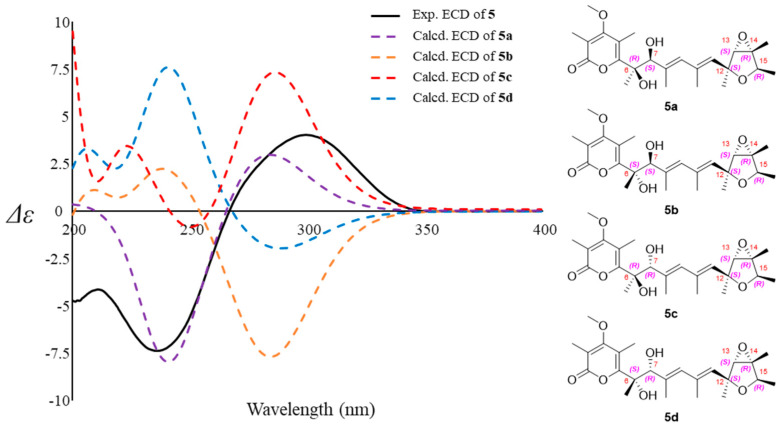
Experimental CD spectra of **5**, the calculated ECD spectra and the structure of **5a**, **5b**, **5c** and **5d** (bandwidth σ = 0.30 eV).

The partial relative configuration of **5** was confirmed by a NOESY experiment (Figure 5). The geometry of C_8_=C_9_ and C_10_=C_11_ were confirmed to be *E* by analysis of the NOESY observations. The key NOESY correlations of H-11 with H-13 and H_3_-22, H_3_-23 with H-13 and H_3_-22, and H-15 with H_3_-21 supported the same relative configurations on furan ring as verrucisidinol [6]. Considering the same biosynthesis origin, compound **5** is deduced to share the same absolute configuration with those of **1**–**4** in the furan ring. In addtion, the optical rotation data of **5** ([α${]}_{D}^{25}$ = +45.0, *c* = 0.1, MeOH) were opposite to that of verrucisidinol ([α${]}_{D}^{25}$ = −10.0, *c* = 0.1, MeOH), implying the enantiomeric relationship between them. To determine the absolute configurations at C-6 and C-7, ECD calculation method was applied. The four configurations (**5a**, **5b**, **5c** and **5d**, Figure 7) were calculated using time-dependent density functional theory (TDDFT) at PBE1PBE/6-311 G* level with PCM model in methanol, and 60 exciting states were calculated. By comparison of the experimental and simulated ECD curves (Figure 7), the experimental ECD was match better with **5a** (6*R*, 7*S*, 12*S*, 13*S*, 14*R*, and 15*R*). Thus, the compound **5** was assigned as 6*R*, 7*S*, 12*S*, 13*S*, 14*R*, and 15*R*, and named as penicicellarusin C.

The molecular formula of penicicellarusin D (**6**) was determined to be C_23_H_32_O_7_ with the unsaturation degrees of eight on the basis of the HRESIMS data at *m*/*z* 443.2047 [M+Na]^+^ (calcd. for C_23_H_32_O_7_Na *m*/*z* 443.2040) and NMR data (Table 2). The NMR data of **6** were similar to those of **5** except for the absence of one singlet methyl group. The key HMBC correlations from H-2 (*δ* 5.60) to C-1, C-3 and C-4, as well as the upfield shift of C-2 (*δ* 88.5) confirmed the disappearance of the methyl group on the C-2 position in **6**. Furthermore, Compound **6** showed similar Cotton effects in the experimental CD spectrum with those of **5** (Figure S5), which assigned the absolute configurations of **6** as 6*R*, 7*S*, 12*S*, 13*S*, 14*R*, and 15*R*. It was designated as penicicellarusin D.

Penicicellarusins E-G (compounds **7–9**) were determined to be fatty acid esters of **5** by interpretation of the HRESIMS, 1D and 2D NMR data (Table 3, Figures S6 and S7), and ECD spectra (Figure S8). The MS/MS data of **7–9** confirmed the presence of the fatty acid moiety in their structures. The pseudo molecular ion peaks [M+H]^+^ at *m*/*z* 673.4669 in **7**, *m/z* 699.4819 in **8**, and *m/z* 697.4668 in **9** together with the fragment ion peaks [M+H-C_16_H_32_O_2_]^+^ at *m*/*z* 435.2381 in **7**, [M+H-C_18_H_32_O_2_]^+^ at *m*/*z* 435.2375 in **8**, [M+H-C_18_H_30_O_2_] ^+^ at *m*/*z* 435.2374 in **9** due to the loss of the corresponding fatty acid moiety. To assign the structure of fatty acid moieties, compounds **7–9** was hydrolyzed with alkaline solution followed by methyl esterification. The fatty acid chain in **7–9** was determined to be the palmitic acid, the oleic acid, and the linoleic acid, respectively, by comparison of the retention time and MS spectrum with those of standards by GC-MS analysis (Figure S9). Compounds **7–9** showed similar Cotton effects in the experimental CD spectrum with those of **5** (Figure S8), which assigned their absolute configurations as 6*R*, 7*S*, 12*S*, 13*S*, 14*R*, and 15*R*.

With the help of single-crystal X-ray crystallographic analysis, the 6*R*, 9*R*, 12*S*, 13*S*, 14*R*, and 15*R* absolute configuration of penicyrone A (**10**) was determined. The value of the Flack absolute structure parameter 0.03 (8) was obtained, and a perspective ORTEP plot was shown in Figure 3 (CCDC 2039558). According to X-ray diffraction analysis, the configuration at the C-6 positions in **10** was 6*R*, instead of 6*S* reported in the literature [28]. The structures of other known compounds were determined by comparing spectroscopic data with those in the literature.

**Figure 8:**
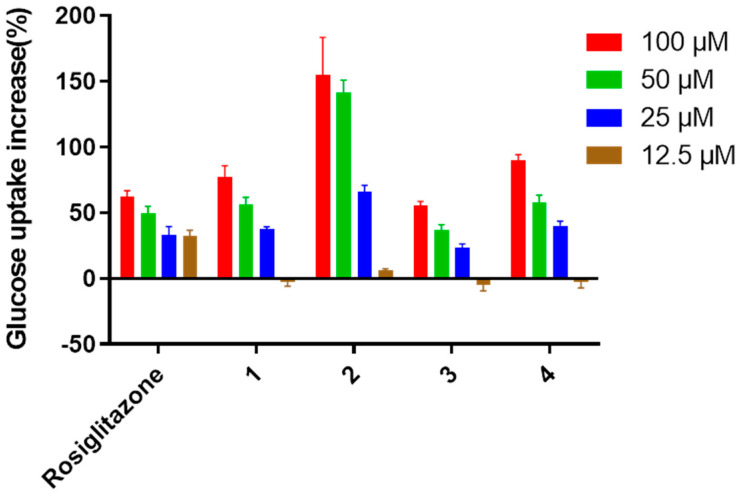
Stimulation on 2-NBDG glucose uptake in insulin-resistant HepG2 cells.

To explore the bioactivities of verrucosidins, compounds **1–12** were evaluated for the antimicrobial effect, cytotoxic activity, and hypoglycemic activity. As a result, **1–12** showed no significant bioactivity in the antimicrobial assays and cytotoxicity assays at the dose of 100 μM. However, Compounds **1–4** were found to enhance the insulin-stimulated uptake of 2-NBDG in insulin-resistant HepG2 cells with the EC_50_ values at 47.2 ± 1.2, 9.9 ± 2.5, 93.2 ± 1.2 and 40.2 ± 1.3 μM, respectively, while the other compounds showed no significant activity (Figure 8). In particular, compounds **1**, **2**, and **4** showed much stronger activity than the positive drug (rosiglitazone) in the range of 25–100 μM.

## 4. Discussion

Up to now, less than 20 verrucosidins and structurally-related compounds have been found in fungi. In this study, seven new verrucosidin derivatives (compounds **3–9**), were isolated together with five previously identified compounds from the fermentation products of fungus *P. cellarum*, suggesting that this fungus is an important producer of verrucosidins.

Verrucosidins share similar structural features with the citreoviridins, including a methylated α-pyrone, a polyene linker, and a tetrahydrofuran ring. The citreoviridin biosynthetic gene cluster containing a polyketide synthase (CtvA), a SAM-dependent methyltransferase (CtvB), a flavin-dependent monooxygenase (CtvC), and a hydrolase (CtvD) has been identified in *Aspergillus terreus var. aureus* [21]. CtvC is the only monooxygenase in the cluster, which can iteratively oxidize the terminal triene portion of the precursor into a bisepoxide moiety. As a regioselective hydrolase, CtvD can transform the bisepoxide moiety into a tetrahydrofuran ring moiety [21,30]. In addition, the verrucosidin bosynthetic gene cluster was confirmed by constructing deletion mutants for *verA* gene coding for HR-PKS known to be the key enzyme of the biosynthesis. Different from citreoviridin, the bosynthetic gene cluster for verrucosidin in the genome of *P. polonicum* contains two flavin-dependent monooxygenases, VerC1 and VerC2, which means that the cluster can synthesize compounds with higher oxidation degree [22]. However, the enzymes involved in the biosynthesis of verrucosidin are largely uncharacterized.

**Figure 9:**
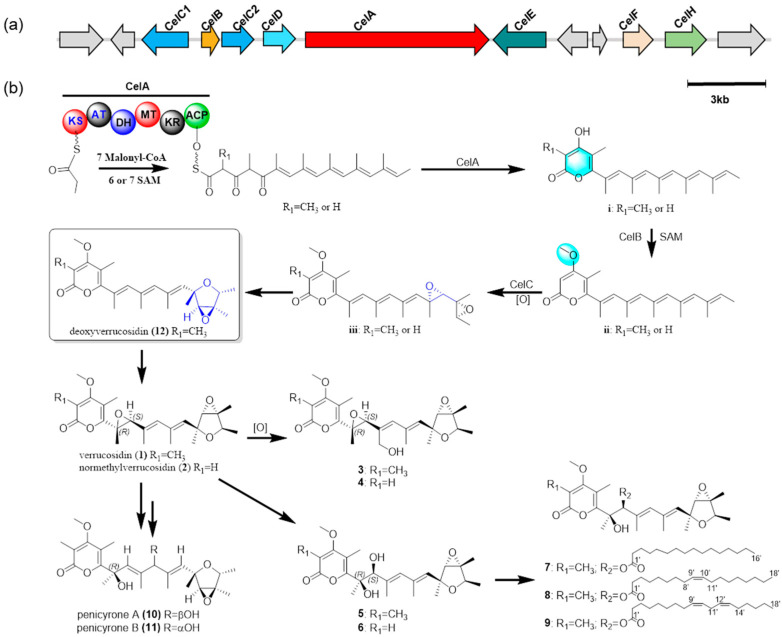
The biosynthetic gene clusters and postulated biogenetic pathway of **1**–**12**. (**a**) The penicicellarusin biosynthesis gene cluster in *P. cellarum*. *celA*: polyketide synthase gene, *celB*: the SAM-dependent methyltransferase gene, *celC1*/*celC2*: the flavin-dependent monooxygenase gene, *celD*: the cytochrome P450 gene, *celF*: the acyl-acyltransferase gene, *celE*: transcriptional factor gene, and *celH*: lyase gene. (**b**) postulated biogenetic pathway of **1**–**12**. PKS domain abbreviations: KS ketosynthase, AT acyltransferase, DH dehydratase, MT methyltransferase, KR keroreductase, ACP acyl carrier protein.

In our work, with the genome of *P. cellarum* YM1 sequenced in our group, we searched for the gene cluster for penicicellarusins. By similarity analysis with the polyketide synthase gene *ctvA* and *verA*, the putative gene cluster *celA* was found in the genome of *P. cellarum* (Table 4 and Figure S11). Further bioinformatic analysis revealed seven genes, the polyketide synthase gene (*celA*), the SAM-dependent methyltransferase gene (*celB*), the flavin-dependent monooxygenase (*celC1 and celC2*), the cytochrome P450 gene (*celD*), the acyl-acyltransferase gene (*celF*), and the lyase gene (*celH*) potentially involved in the biosynthesis of penicicellarusins in *P. cellarum*. Based on above evidence, we propose the biosynthetic pathway of **1–12** (Figure 9 and Figure S10). Verrucosidin (**1**) could be formed from **12** by oxidation of the olefinic bond, and further oxidation produces **3–6**. Compounds **7–9** were biosynthesized by esterification of **5** with different fatty acids. Compounds **10** and **11** can be transformed from **5** or **6** by dehydration reaction.

Early studies have demonstrated that verrucosidins and structurally-related compounds are endowed with several interesting bioactivities, such as antibacterial activities [3,10], antitumor [7,8], antiviral [9], and neurological activities [11]. In this work, it was found that compounds **1**–**4** show promising hypoglycemic activity, especially compounds **2** and **4**. Preliminary structure-activity relationship showed that the formation of epoxy three-membered ring on C_6_-C_7_ in the structures contributes greatly for the glucose uptake-enhancing activity in insulin-resistant HepG2 cells. The promising hypoglycemic activity is an interesting new bioactivity for this class of compounds.

## 5. Conclusions

In summary, a MS/MS-based molecular networking for the target discovery of verrucosidin-like polyketides was established in this study. The stereochemistry of the new compounds was determined by electronic circular dichroism (ECD) methods or comparison of experimental ECD spectra. The absolute configuration of penicyrone A (**10**) was corrected based on X-ray diffraction analyses. Bioactivity screening indicated that compounds **1**, **2**, and **4** showed much stronger promising hypoglycemic activity than the positive drug (rosiglitazone) in the range of 25–100 μM. The promising hypoglycemic activity is an interesting new bioactivity for this class of compounds. This work further proved the efficacy of the molecular networking in discovering natural products with unique structural features.

## Figures and Tables

**Table 1:** ^1^H and ^13^C-NMR Data for compounds **3–4** in CD_3_OD.

Pos.	3		4		Pos.	3		4	
	*δ*_C_	*δ*_H_ (*J* in Hz)	*δ*_C_	*δ*_H_ (*J* in Hz)		*δ*_C_	*δ*_H_ (*J* in Hz)	*δ*_C_	*δ*_H_ (*J* in Hz)
1	167.5		166.5		14	68.7		68.7	
2	111.6		89.5	5.66 s	15	78.4	4.09 q (6.8)	78.4	4.09 q (6.8)
3	170.2		173.2		16	10.4	2.04 s		
4	112.2		109.9		17	9.7	2.12 s	8.9	2.07 s
5	157.3		159.3		18	15.7	1.44 s	15.6	1.44 s
6	62.2		62.2		19	59.6	4.36 d (12.2)	59.6	4.36 d (12.2)
7	64.6	3.81 s	64.5	3.81 s			4.41 d (12.2)		4.40 d (12.2)
8	133.5		133.5		20	18.7	1.98 s	18.7	1.98 s
9	134.9	5.98 brs	134.9	5.98 brs	21	22.1	1.40 s	22.1	1.40 s
10	135.6		135.6		22	13.8	1.48 s	13.8	1.48 s
11	133.9	5.60 brs	133.9	5.60 brs	23	19.2	1.23 d (6.8)	19.2	1.23 d (6.8)
12	81.4		81.4		24	61.3	3.91 s	57.4	3.92 s
13	68.7	3.60 s	68.7	3.59 s					

**Table 2:** ^1^H and ^13^C-NMR Data for compounds **5–6**.

Pos.	Verrucisidinol ^a,c^		5 ^a^		5 ^b^		6 ^b^	
	*δ*_C_	*δ*_H_ (*J* in Hz)	*δ*_C_	*δ*_H_ (*J* in Hz)	*δ*_C_	*δ*_H_ (*J* in Hz)	*δ*_C_	*δ*_H_ (*J* in Hz)
1	165.0		165.5		166.1		166.6	
2	110.4		110.2		109.4		88.5	5.60 s
3	169.2		170.4		170.3		174.3	
4	111.8		112.7		112.4		111.5	
5	159.7		160.9		160.6		163.8	
6	78.8		79.1		78.9		81.4	
7	79.8	4.61 s	79.3	4.73 s	79.8	4.34 s	80.4	4.35 s
8	133.9		134.1		132.5		133.9	
9	134.3	5.87 s	134.5	5.91 brs	135.1	5.69 brs	136.5	5.71 brs
10	134.4		134.6		135.2		136.6	
11	133.0	5.43 s	133.0	5.43 brs	131.4	5.42 brs	132.8	5.43 brs
12	80.1		80.3		80.7		82.1	
13	67.5	3.43 s	67.6	3.43 s	67.3	3.54 s	68.8	3.55 s
14	67.4		67.5		67.2		68.7	
15	76.7	4.12 q (7.0)	76.8	4.12 q (6.8)	76.8	4.05 q (6.8)	78.3	4.06 q (6.8)
16	10.2	2.01 s	10.3	2.04 s	8.8	2.00 s		
17	9.9	2.21 s	10.1	2.22 s	8.9	2.28 s	9.4	2.25 s
18	23.4	1.40 s	23.6	1.40 s	21.5	1.48 s	22.9	1.49 s
19	14.8	1.82 d	14.9	1.83 d	13.5	1.82 s	14.9	1.83 s
20	18.6	1.89 s	18.7	1.88 s	17.4	1.86 s	18.8	1.87 s
21	21.9	1.41 s	22.0	1.41 s	20.7	1.37 s	22.1	1.38 s
22	13.8	1.47 s	14.0	1.46 s	12.4	1.47 s	13.8	1.48 s
23	18.8	1.18 d (7.0)	19.0	1.18 d (6.8)	17.8	1.17 d (6.8)	19.2	1.19 d (6.8)
24	60.3	3.79 s	60.6	3.79 s	59.6	3.84 s	57.3	3.90 s

^a^ NMR data were measured in CD_3_Cl; ^b^ NMR data were measured in CD_3_OD; ^c^ NMR data reported in literature.

**Table 3:** ^1^H and ^13^C-NMR Data for compounds **7–9** in CD_3_OD.

Pos.	7		8		9	
	*δ*_C_	*δ*_H_ (*J* in Hz)	*δ*_C_	*δ*_H_ (*J* in Hz)	*δ*_C_	*δ*_H_ (*J* in Hz)
1	167.0		167.0		167.0	
2	111.2		111.2		111.2	
3	171.3		171.3		171.3	
4	114.2		114.2		114.2	
5	160.4		160.4		160.4	
6	79.3		79.3		79.3	
7	83.8	5.39 s	83.8	5.39 s	83.3	5.39 s
8	132.8		132.8		132.8	
9	135.8	5.79 brs	135.8	5.79 brs	135.8	5.79 brs
10	136.2		136.2		136.2	
11	133.5	5.43 brs	133.5	5.43 brs	133.5	5.43 brs
12	81.4		81.4		81.4	
13	68.7	3.54 s	68.7	3.54 s	68.7	3.54 s
14	68.7		68.7		68.7	
15	78.3	4.05 q (6.8)	78.3	4.05 q (6.8)	78.2	4.06 q (6.8)
16	10.3	2.01 s	10.3	2.01 s	10.3	2.01 s
17	10.4	2.28 s	10.4	2.28 s	10.4	2.29 s
18	23.7	1.56 s	23.8	1.56 s	23.6	1.56 s
19	15.9	1.83 s	15.9	1.83 s	15.9	1.83 s
20	18.7	1.87 s	18.7	1.87 s	18.8	1.87 s
21	22.1	1.36 s	22.1	1.36 s	22.1	1.36 s
22	13.8	1.47 s	13.8	1.47 s	13.8	1.47 s
23	19.2	1.18 d (6.8)	19.2	1.18 d (6.8)	19.2	1.18 d (6.8)
24	61.1	3.84 s	61.1	3.84 s	61.1	3.84 s
1’	174.2		174.2		174.1	
2’	35.2	2.35 dt (2.4, 7.3)	35.2	2.35 dt (2.3, 7.3)	35.2	2.35 dt (2.4, 7.3)
3’	26.1	1.59 m	26.1	1.59 m	26.1	1.59 m
9’			130.8	5.36 m	130.8	5.36 m
10’			130.9	5.36 m	130.9	5.36 m
12’					129.0	5.36 m
13’					129.1	5.36 m
16’	14.5	0.92 t (6.8)				
18’			14.5	0.91 t (6.8)	14.5	0.92 t (6.8)
Others	29.1–30.9	1.30 m	29.1–30.9	1.30 m	29.1–30.9	1.30 m

“m” means multiplet or overlapped with other signals.

**Table 4:** Penicicellarusins biosynthetic genes and gene function prediction in *P. cellarum* and their homologs in other fungal species.

*P. cellarum* Gene	*P. polonicum* Homologue aa (Identity/Similarity, %), Gene (Identity/Similarity, %)	*A. terreus* Homologue aa (Identity/Similarity, %)	Putative Function
celA	VerA (86/98) PENPOL_C002G03804 (99/98)	CtvA (42/77)	Polyketide synthase
celB	VerB (79/29) PENPOL_C002G01780 (98/41)	CtvB (30/41)	Methyltransferase
celC1	VerC1 (99/93) PENPOL_c002G07872 (99/45)	CtvC (50/56)	FAD monooxygenase
celC2	VerC2 (91/50) PENPOL_C002G07909 (99/57)	-	FAD monooxygenase
celD	VerH (99/95) PENPOL_c002G07307 (98/69)	-	Cytochrome P450
celE	VerF (78/96) PENPOL_c002G07024 (99/95)	-	Transcription factor domain
celF	VerG (87/100) PENPOL_c002G05667 (97/99)	-	Acyl-acyltransferase
celH	-	-	Lyase

## Data Availability

Not applicable.

## Supplementary Materials

The following are available online at https://www.mdpi.com/article/10.3390/jof8020143/s1, Figure S1: The HPLC profiles of metabolites extracted from the culture medium of *Penicillium* strains, Figure S2: The molecular network obtained by combining the LC-MS/MS analyses of extracts from *P. cellarum* YM1, Figure S3: Phylogenetic analysis and morphological characters of *P. cellarum* YM1, Figure S4: Most stable conformers of **5** in solvated model calculations at the B3LYP/6-31+G(d,p) level (d), Figure S5: Experimental CD spectra of **5** and **6** in MeOH, Figure S6: Selected key HMBC and ^1^H-^1^H COSY correlations of **7–9**, Figure S7: Selected key NOE correlations of **7–9**, Figure S8: Experimental CD spectra of **5** and **7–9** in MeOH, Figure S9: GC-MS analysis of methyl linoleate, methyl oleate and products of alkaline hydrolysis-methyl esterification of compounds **8** and **9**, Figure S10: Gene cluster schematic illustrating comparative organization of the penicicellarusin, verrucosidin, and citreoviridin, Figures S11–S40: NMR spectra for compounds **3–9**.
