# Supplementary material for: Exploring Verrucosidin Derivatives with Glucose-Uptake-Stimulatory Activity from Penicillium cellarum Using MS/MS-Based Molecular Networking
Source: J Fungi (Basel). 2022 Jan 30;8(2):143. doi: 10.3390/jof8020143 (PMC8878765; doi:10.3390/jof8020143)
Supplement: Supplementary file 1 [file jof-08-00143-s001.zip › jof-1563123-supplementary.pdf]

Supporting Information

Exploring verrucosidin derivatives with glucose-uptake-stimulatory activity from *Penicillium cellarum* using MS/MS-based molecular networking

Junjie Han, Baosong Chen, Rui Zhang, Jinjin Zhang, Huanqin Dai, Tao Wang, Jingzu Sun, Guoliang Zhu, Wei Li, Erwei Li, Xueting Liu, Wenbing Yin, and Hongwei Liu*

Contents

**Figure S1**	The HPLC profiles of metabolites extracted from the culture medium of *Penicillium* strains.	Page S4
**Figure S2**	The molecular network obtained by combining the LC-MS/MS analyses of extracts from *P. cellarum* YM1.	Page S5
**Figure S3**	Phylogenetic analysis and morphological characters of *P. cellarum* YM1.	Page S6
**Figure S4**	Most stable conformers of **5a**, **5b**, **5c**, **5d** in solvated model calculations at the B3LYP/6-31+G(d,p) level (d) (the relative populations are in parentheses)	Page S7
**Figure S5**	Experimental CD spectra of **5** and **6** in MeOH.	Page S8
**Figure S6**	Selected key HMBC and ^{1}H-^{1}H COSY correlations of **7-9.**	Page S9
**Figure S7**	Selected key NOE correlations of **7-9**.	Page S10
**Figure S8**	Experimental CD spectra of **5** and **7-9** in MeOH.	Page S11
**Figure S9**	GC-MS analysis of methyl linoleate, methyl oleate and products of alkaline hydrolysis-methyl esterification of compounds **8** and **9.**	Page S12
**Figure S10**	Gene cluster schematic illustrating comparative organization of the penicicellarusin, verrucosidin, and citreoviridin	Page S13
**Figure S11**	^{1}H NMR spectrum of penicicellarusin C (**3**) in CD_3OD (500 MHz).	Page S14
**Figure S12**	^{13}C NMR spectrum of penicicellarusin C (**3**) in CD_3OD (125 MHz).	Page S15
**Figure S13**	HMBC spectrum of penicicellarusin C (**3**) in CD_3OD.	Page S16
**Figure S14**	NOESY spectrum of penicicellarusin C (**3**) in CD_3OD.	Page S17
**Figure S15**	^{1}H NMR spectrum of penicicellarusin D (**4**) in CD_3OD (500 MHz).	Page S18
**Figure S16**	^{13}C NMR spectrum of penicicellarusin D (**4**) in CD_3OD (125 MHz).	Page S19
**Figure S17**	HMBC spectrum of penicicellarusin D (**4**) in CD_3OD.	Page S20
**Figure S18**	NOESY spectrum of penicicellarusin D (**4**) in CD_3OD.	Page S21
**Figure S19**	^{1}H NMR spectrum of penicicellarusin E (**5**) in $CDCl_3$ (500 MHz).	Page S22
**Figure S20**	^{13}C NMR spectrum of penicicellarusin E (**5**) in $CDCl_3$ (125 MHz).	Page S23
**Figure S21**	^{1}H NMR spectrum of penicicellarusin E (**5**) in CD_3OD (500 MHz).	Page S24
**Figure S22**	^{13}C NMR spectrum of penicicellarusin E (**5**) in CD_3OD (125 MHz).	Page S25
**Figure S23**	HMBC spectrum of penicicellarusin E (**5**) in CD_3OD.	Page S26
**Figure S24**	NOESY spectrum of penicicellarusin E (**5**) in CD_3OD.	Page S27
**Figure S25**	^{1}H NMR spectrum of penicicellarusin F (**6**) in CD_3OD (500 MHz).	Page S28
**Figure S26**	^{13}C NMR spectrum of penicicellarusin F (**6**) in CD_3OD (125 MHz).	Page S29
**Figure S27**	HMBC spectrum of penicicellarusin F (**6**) in CD_3OD.	Page S30
**Figure S28**	NOESY spectrum of penicicellarusin F (**6**) in CD_3OD.	Page S31
**Figure S29**	^{1}H NMR spectrum of penicicellarusin G (**7**) in CD_3OD (500 MHz).	Page S32
**Figure S30**	^{13}C NMR spectrum of penicicellarusin G (**7**) in CD_3OD (125 MHz).	Page S33
**Figure S31**	HMBC spectrum of penicicellarusin G (**7**) in CD_3OD.	Page S34
**Figure S32**	NOESY spectrum of penicicellarusin G (**7**) in CD_3OD.	Page S35
**Figure S33**	^{1}H NMR spectrum of penicicellarusin H (**8**) in CD_3OD (500 MHz).	Page S36
**Figure S34**	^{13}C NMR spectrum of penicicellarusin H (**8**) in CD_3OD (125 MHz).	Page S37
**Figure S35**	HMBC spectrum of penicicellarusin H (**8**) in CD_3OD.	Page S38
**Figure S36**	NOESY spectrum of penicicellarusin H (**8**) in CD_3OD.	Page S39

**Figure S37**	^{1}H NMR spectrum of penicicellarusin I (**9**) in CD_3OD (500 MHz).	Page S40
**Figure S38**	^{13}C NMR spectrum of penicicellarusin I (**9**) in CD_3OD (125 MHz).	Page S41
**Figure S39**	HMBC spectrum of penicicellarusin I (**9**) in CD_3OD.	Page S42
**Figure S40**	NOESY spectrum of penicicellarusin I (**9**) in CD_3OD.	Page S43

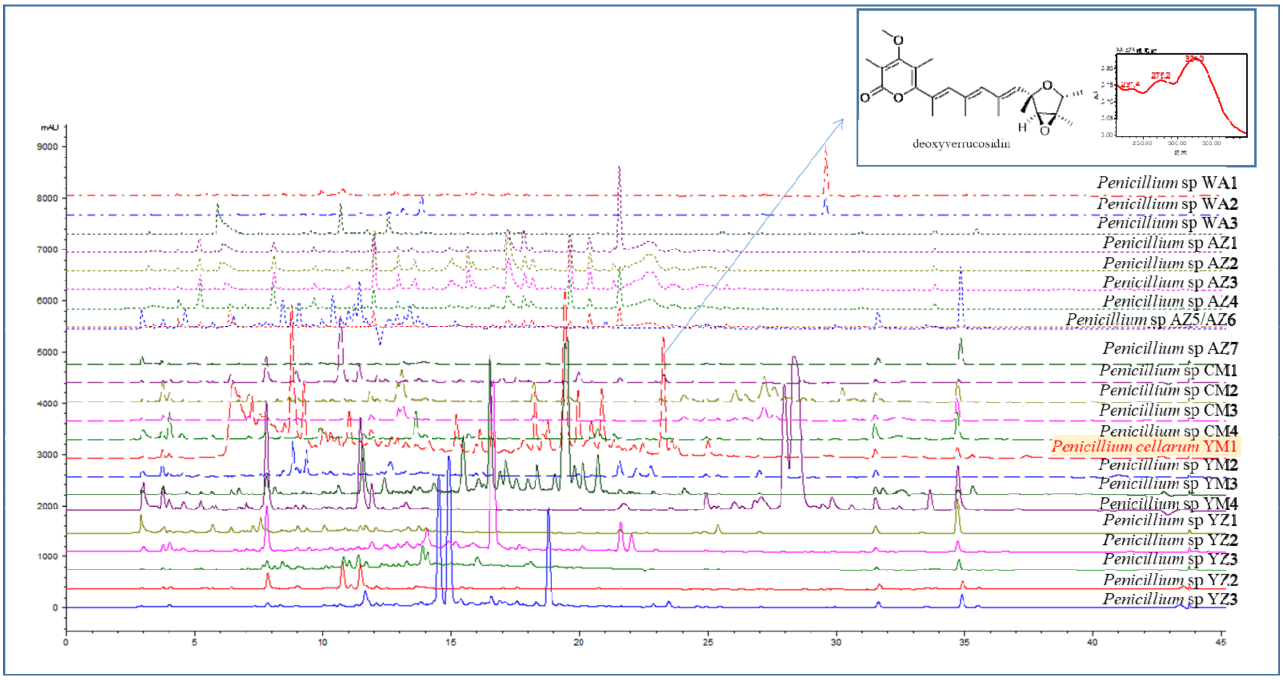

Figure S1. The HPLC profiles of metabolites extracted from the culture medium of *Penicillium* strains. HPLC analysis was carried out using a C18 reversed-phase column. Detection was at 235 nm.

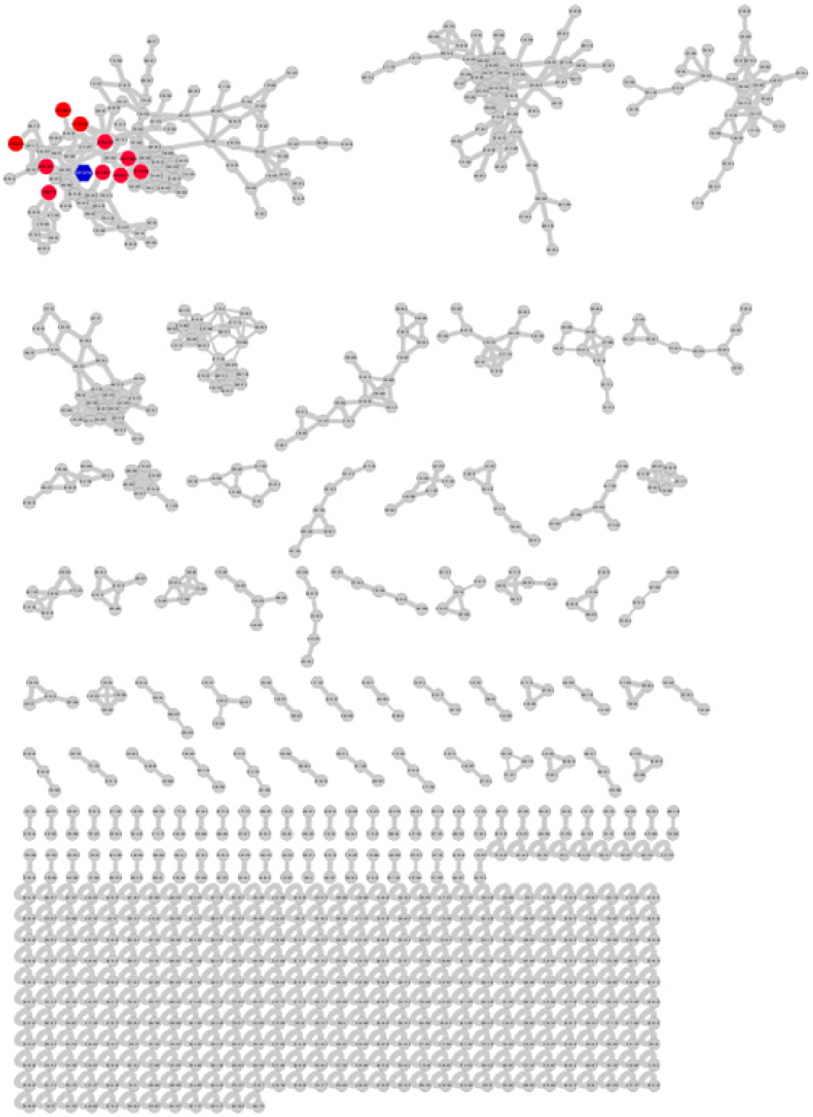

Figure S2. The molecular network obtained by combining the LC-MS/MS analyses of extracts from *P. cellarum*
https://gnps.ucsd.edu/ProteoSAFe/status.jsp?task=8716192add914a1fb3bd8f469f7d2d81

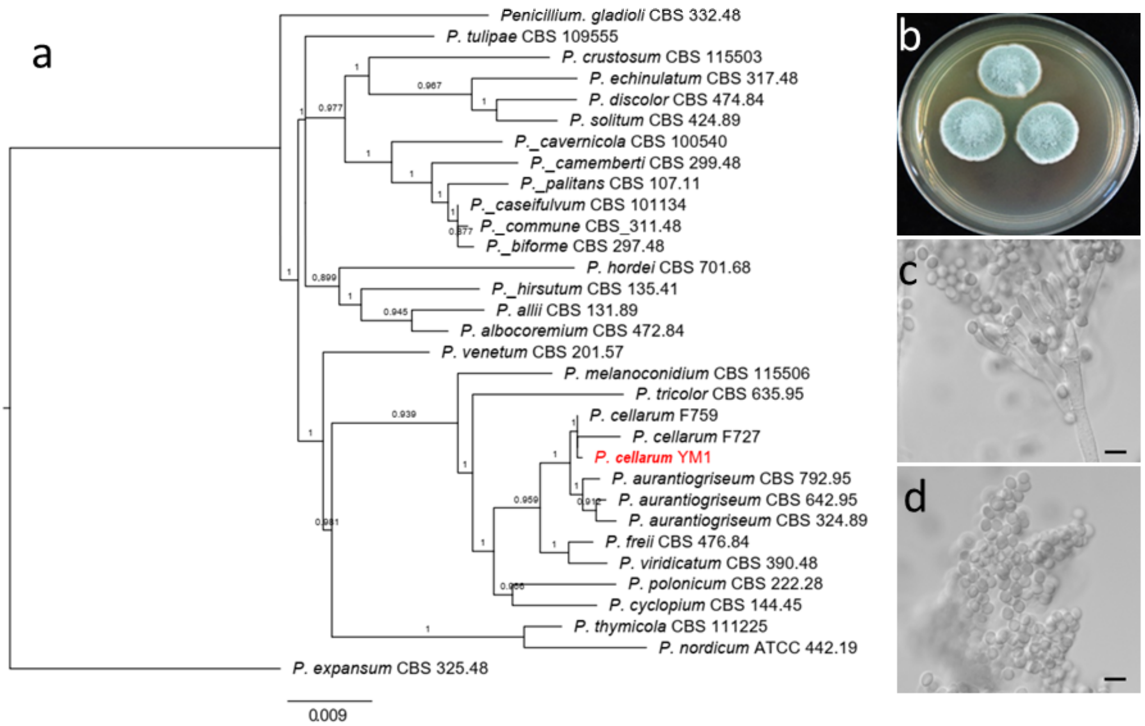

Figure S3. Phylogenetic analysis and morphological characters of *P. cellarum*

a. Phylogenetic analysis of *P. cellarum* based on BenA-RPB2-CaM dataset. b. colony on PDA after 7 d; c-d. conidiophores and conidia. Bars in c-d = 5 μm.

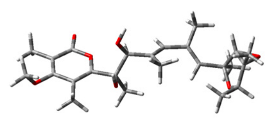

5a-1 37.57%

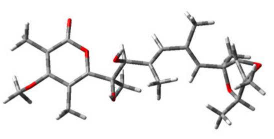

5a-2 23.32%

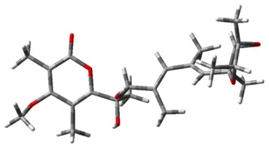

5a-3 15.58%

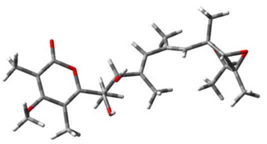

5a-4 13.74%

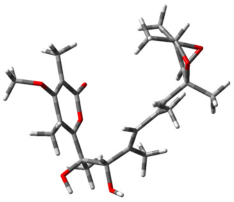

5a-5 5.44%

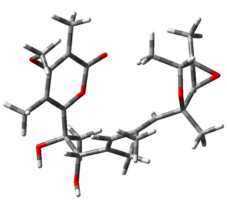

5a-6 4.34%

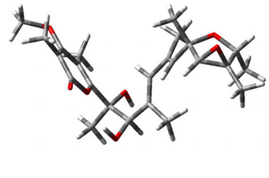

5b-1 19.55%

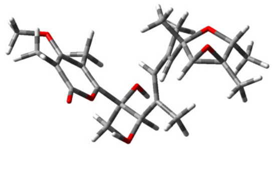

5b-2 19.54%

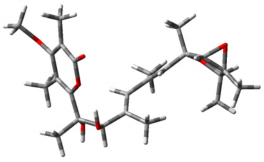

5b-3 19.22%

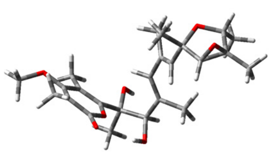

5b-4 16.90%

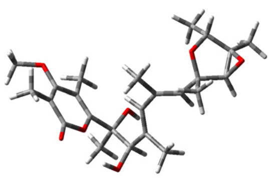

5b-5 16.73%

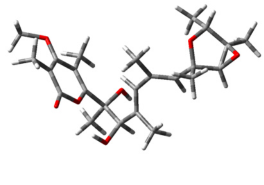

5b-6 8.05%

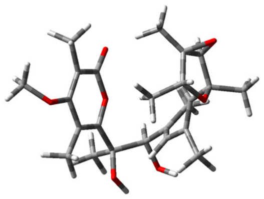

5c-1 57.73%

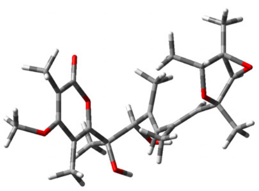

5c-2 42.26%

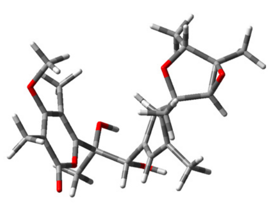

5d-1 17.90%

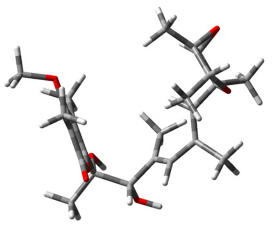

5d-2 17.89%

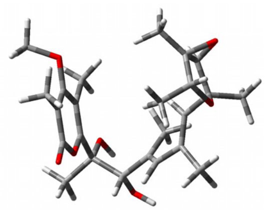

5d-3 17.89%

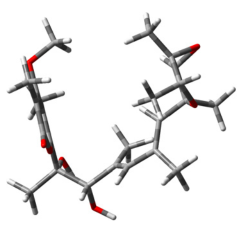

5d-4 17.89%

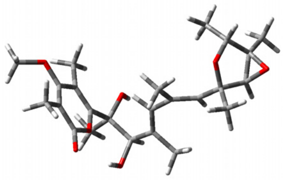

5d-5 9.46%

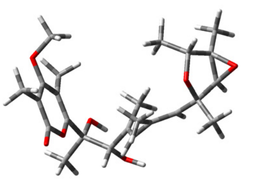

5d-6 9.46%

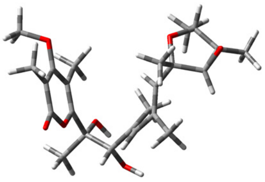

5d-7 9.46%

Figure S4. Most stable conformers of **5a**, **5b**, **5c**, **5d** in solvated model calculations at the B3LYP/6-31+G(d,p) level (d) (the relative populations are in parentheses).

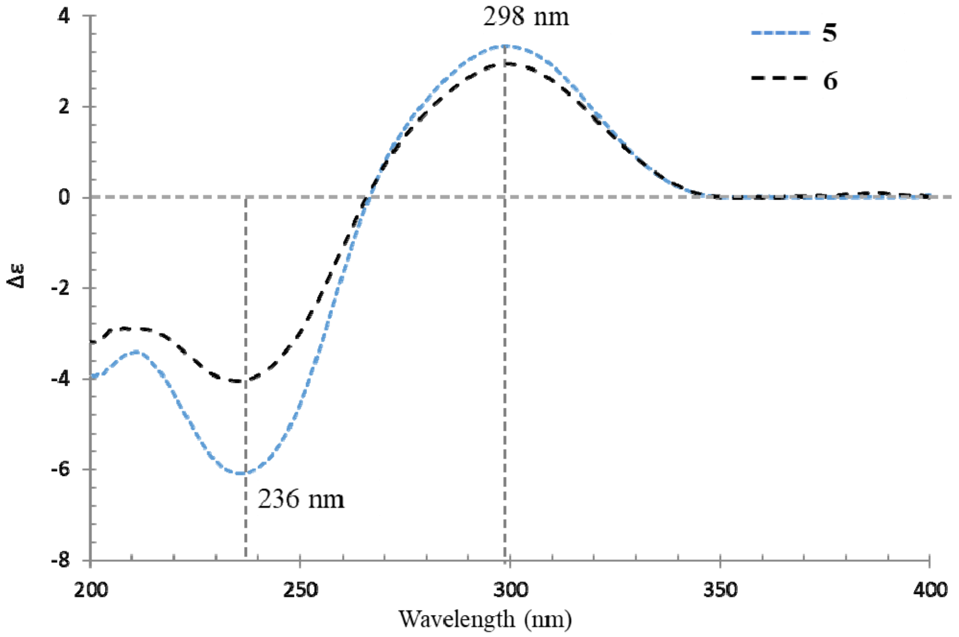

Figure S5. Experimental CD spectra of **5** and **6** in MeOH

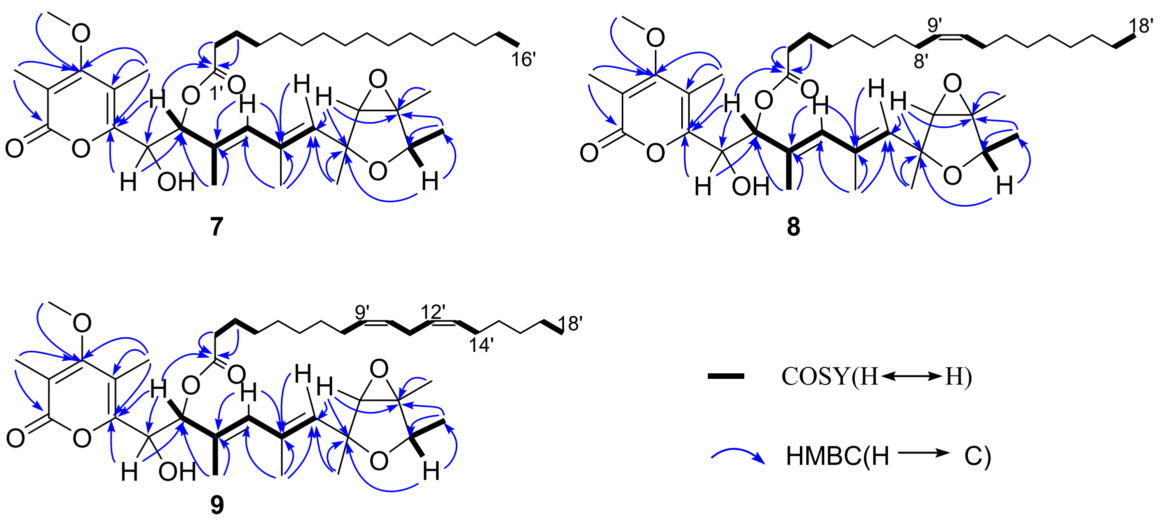

Figure S6. Selected key HMBC and ^{1}H-^{1}H COSY correlations of **7-9.**

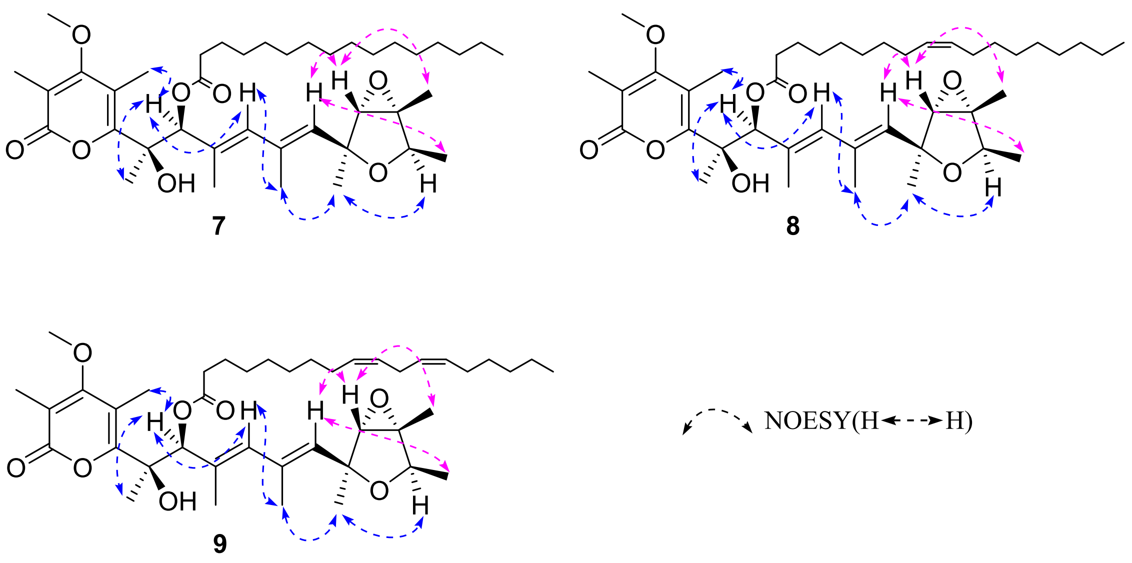

Figure S7. Selected key NOE correlations of **2-4**, **6**, and **7-9**.

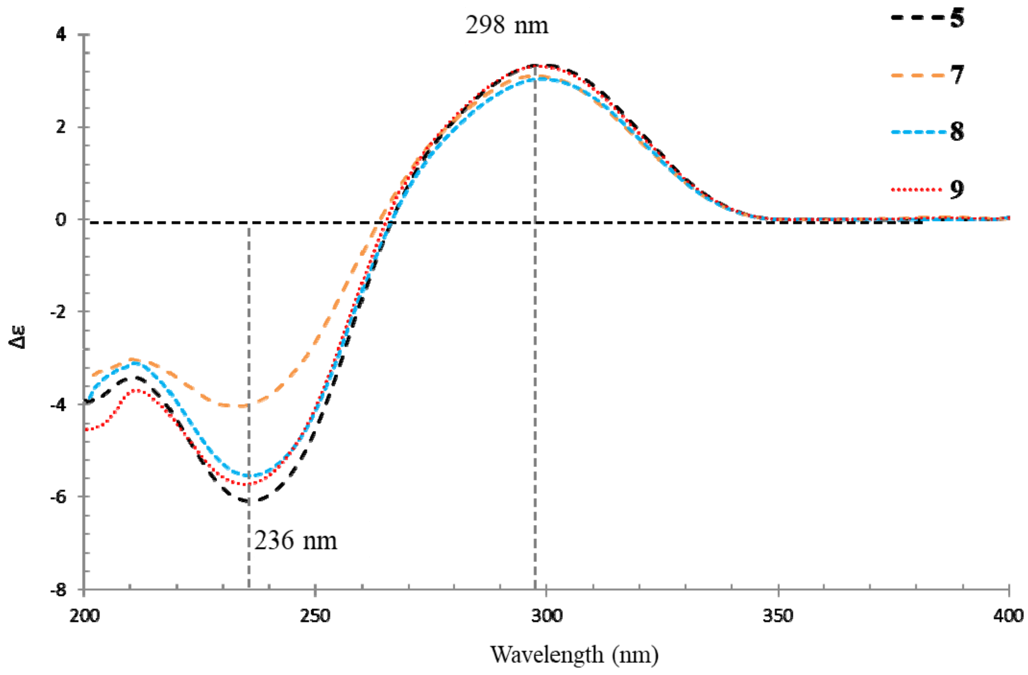

Figure S8. Experimental CD spectra of **5** and **7-9** in MeOH

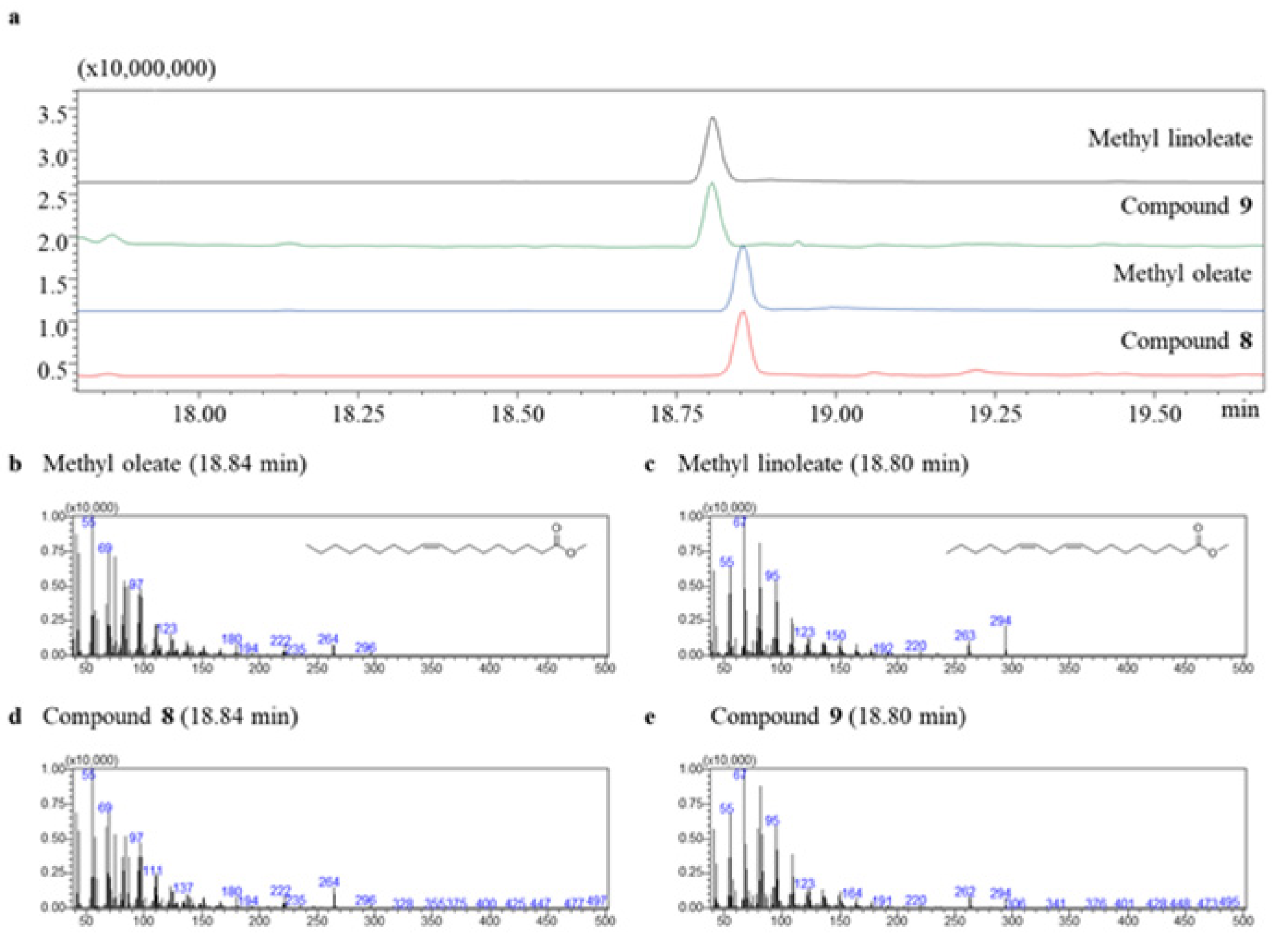

Figure S9. GC-MS analysis of methyl linoleate, methyl oleate and products of alkaline hydrolysis-methyl esterification of compounds **8** and **9**.

The samples were analyzed in split injector mode by using a fused silica capillary column Rtx-5MS (crosslinked 5% diphenyl dimethyl polysiloxane, 30 m × 0.25 mm ID × 0.25 μM) with helium (1 mL/min) as carrier. Oven temperature was programmed from 50 ºC to 325 ºC at a slope of 10 ºC per minute and then with 15 minutes hold. The MS was operated in EI mode (70 eV) scanning from 40 to 500 amu. The retention time of methyl oleate and methyl linoleate were 18.80 and 18.84 min, separately. (a) GC chromatogram of methyl linoleate, methyl oleate and products of alkaline hydrolysis-methyl esterification of compounds **8** and **9**; (b-e) mass spectrogram of methyl linoleate, methyl oleate and products of alkaline hydrolysis-methyl esterification of compounds **8** and **9**, separately.

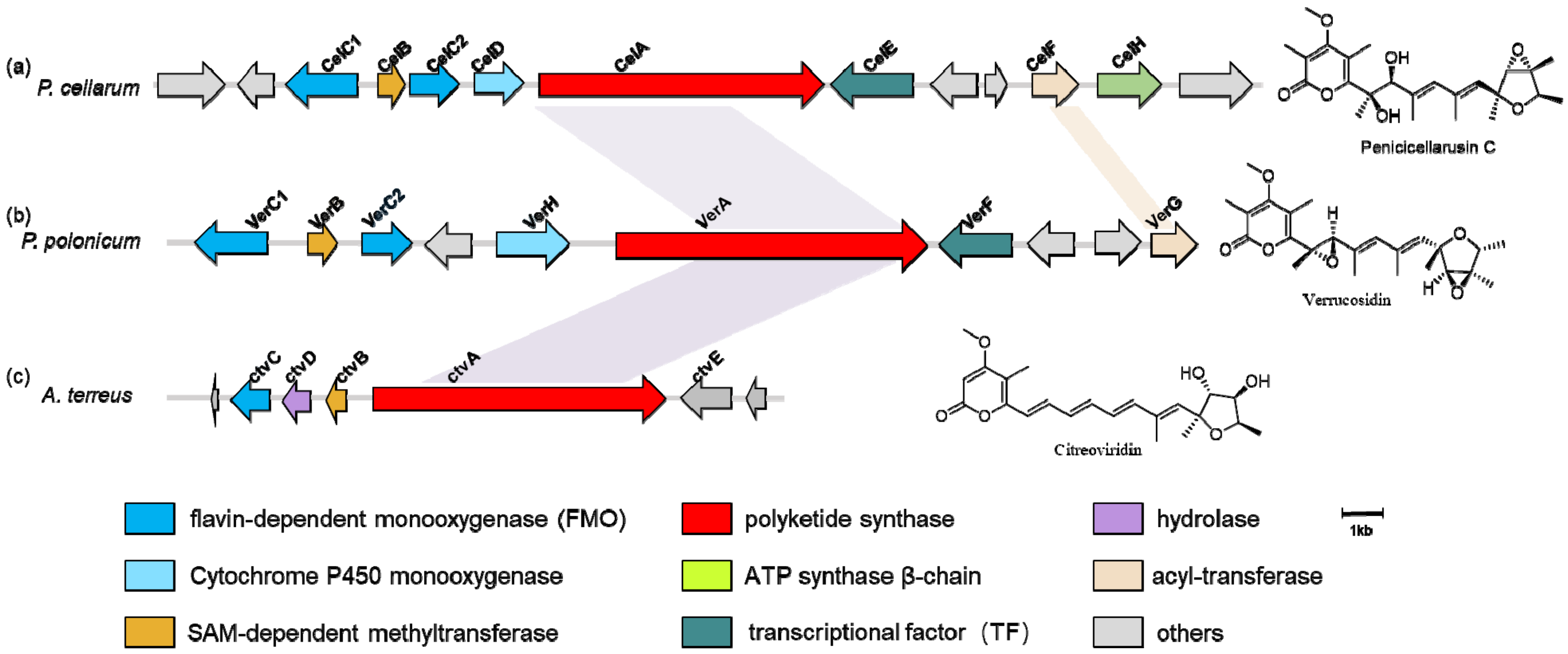

Figure S10. Gene cluster schematic illustrating comparative organization of the penicicellarusin, verrucosidin, and citreoviridin. (**a**) Penicicellarusin gene cluster in *P. cellarum*. (**b**) Verrucosidin gene cluster in *P. polonicum*. (**c**) Citreoviridin gene cluster in *A. terreus*.

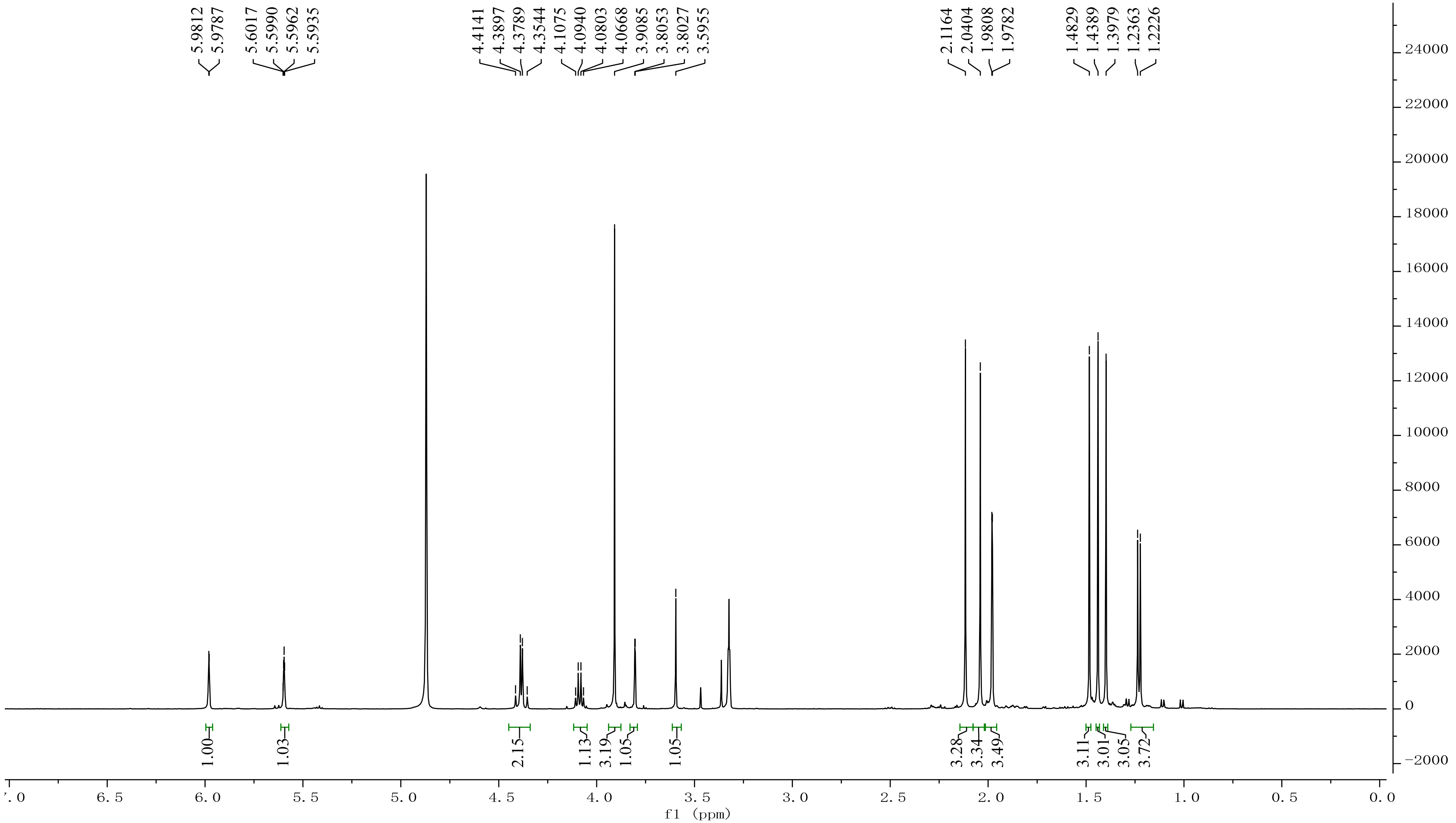

Figure S11. ^1H NMR spectrum of penicicellarusin A (**3**) in CD_3OD (500 MHz)

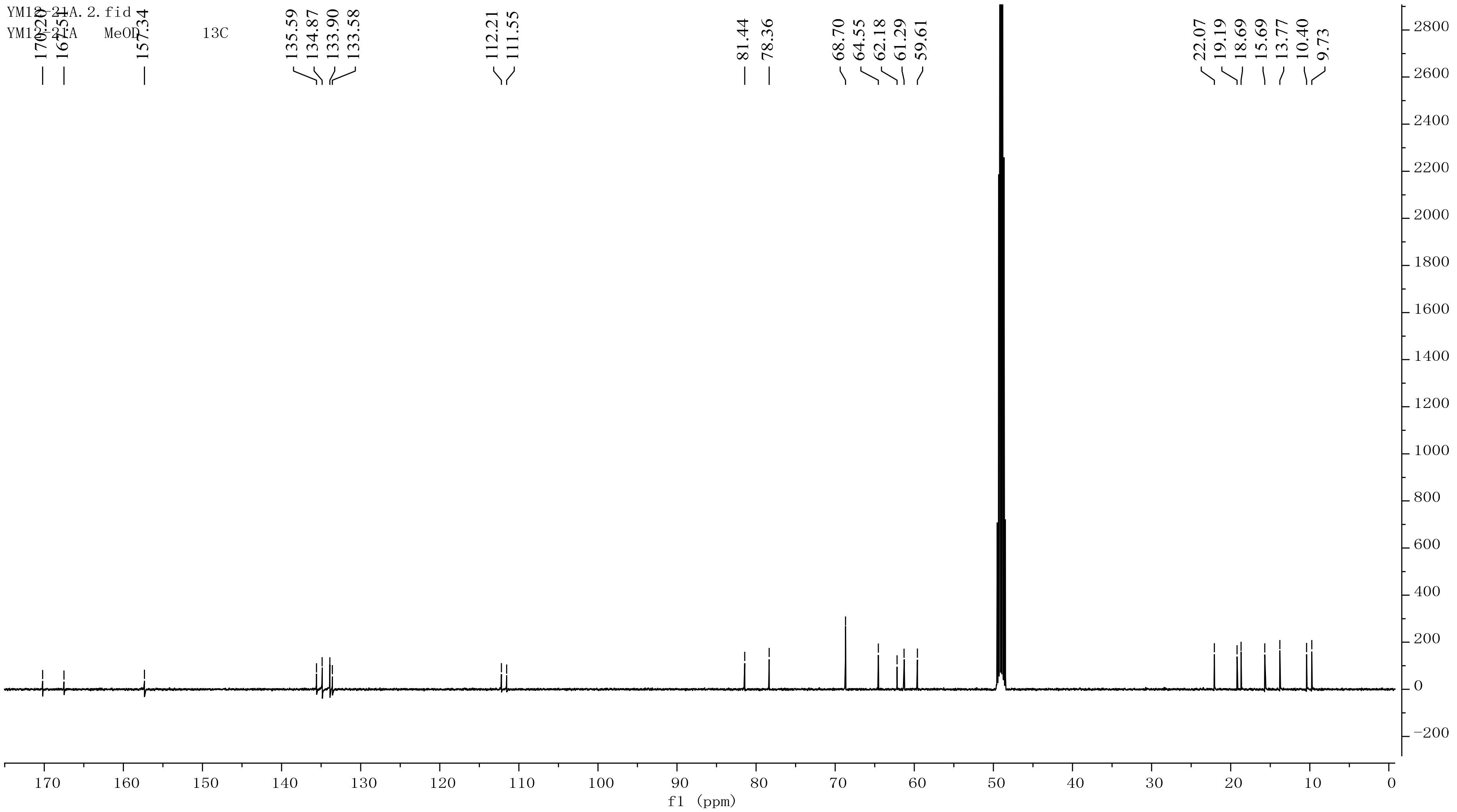

Figure S12. ^{13}C NMR spectrum of penicicellarusin A (**3**) in CD_3OD (125 MHz)

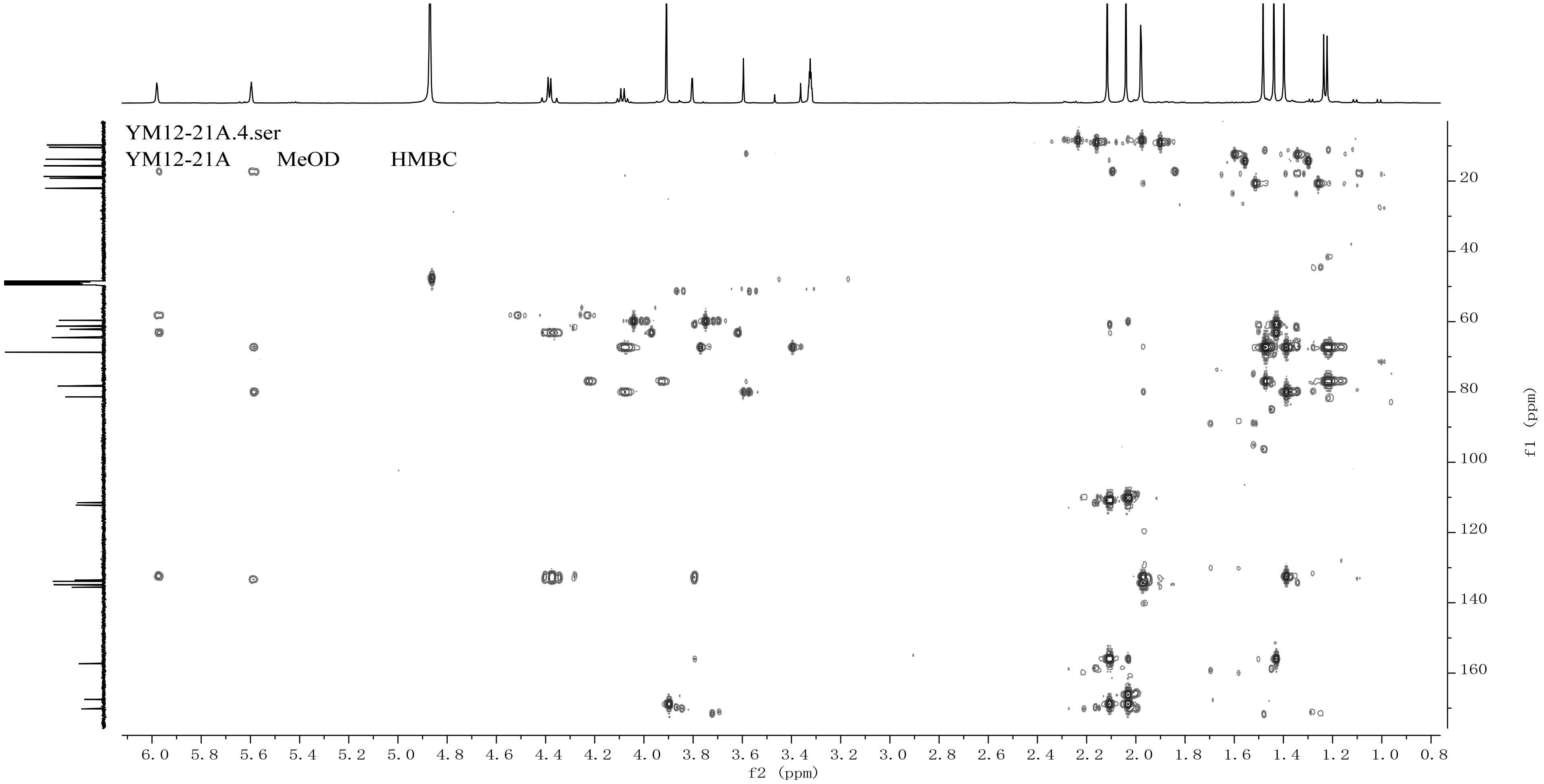

Figure S13. HMBC spectrum of penicicellarusin C (**3**) in CD_3OD

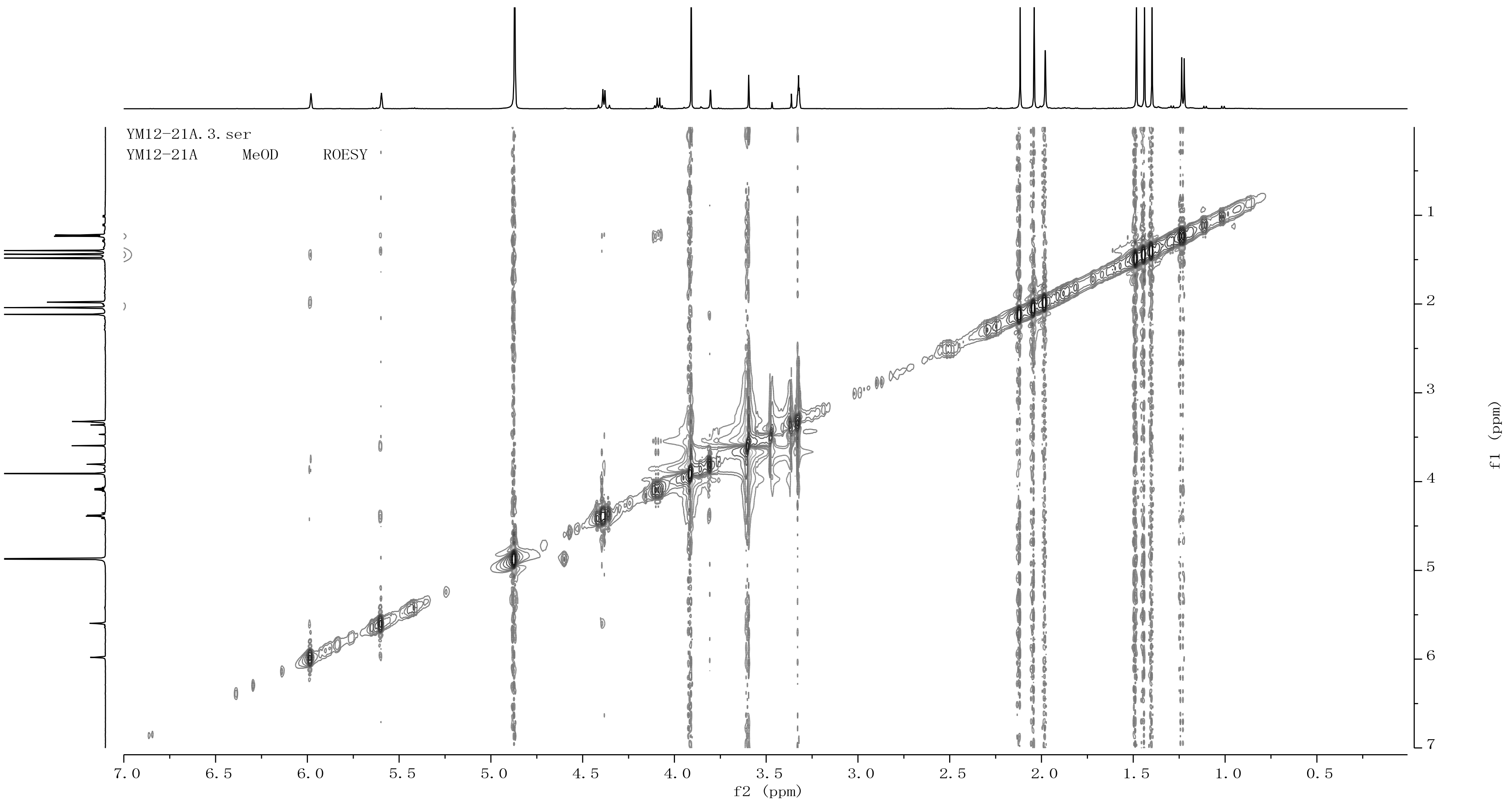

Figure S14. NOESY spectrum of penicicellarusin A (**3**) in CD_3OD

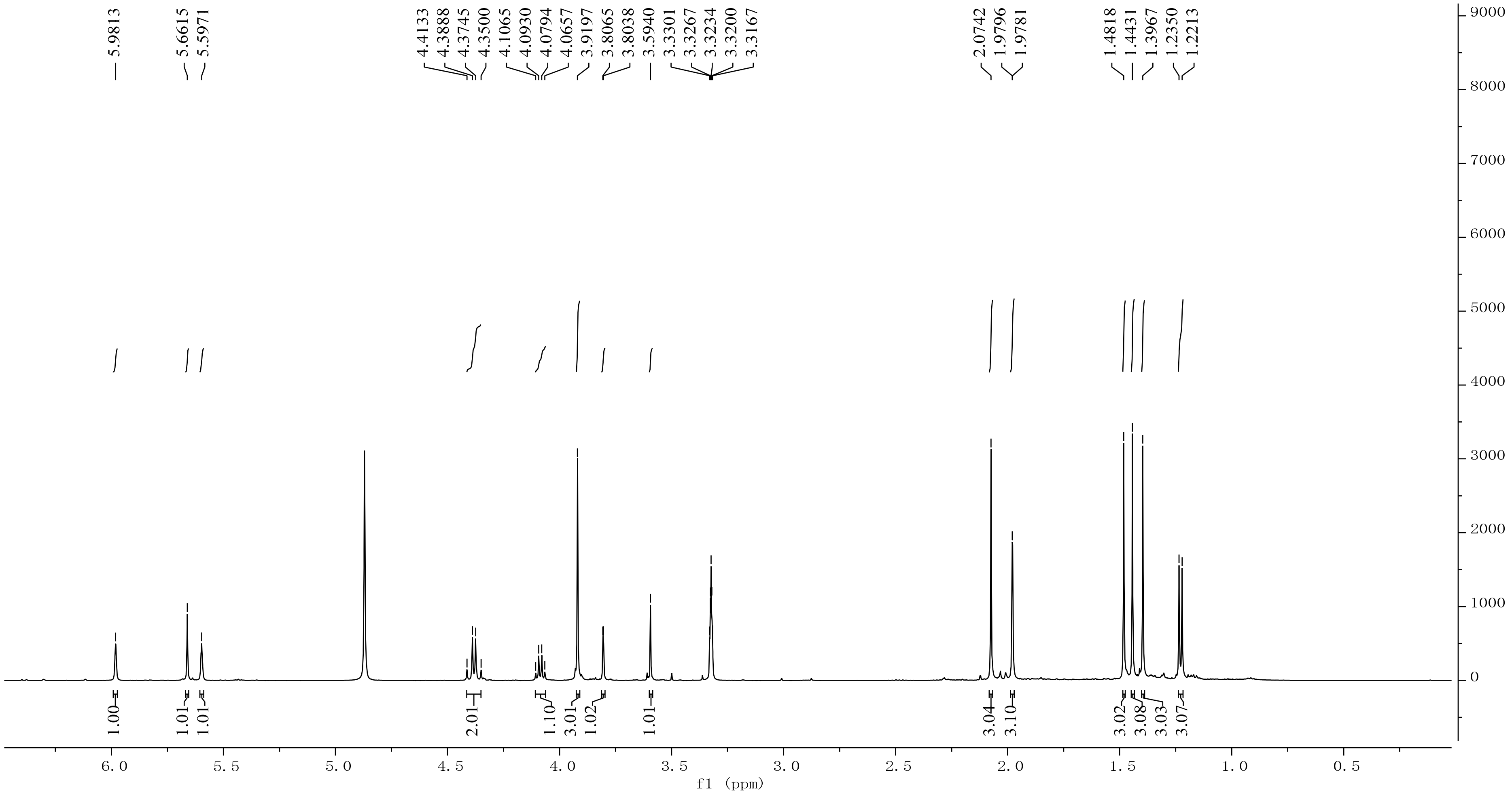

Figure S15. ^{1}H NMR spectrum of penicicellarusin B (**4**) in CD_3OD (500 MHz)

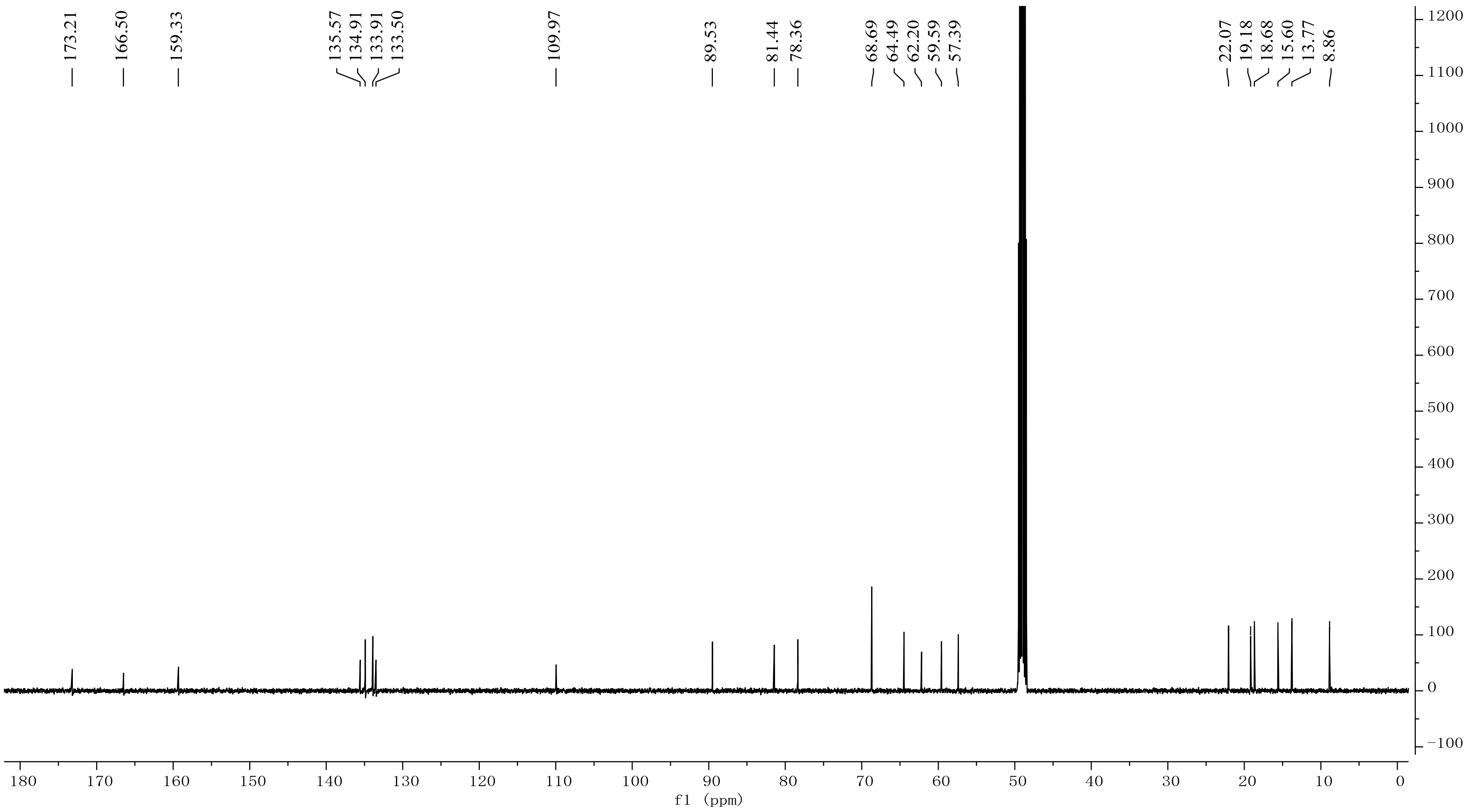

Figure S16. ^{13}C NMR spectrum of penicicellarusin B (**4**) in CD_3OD (125 MHz)

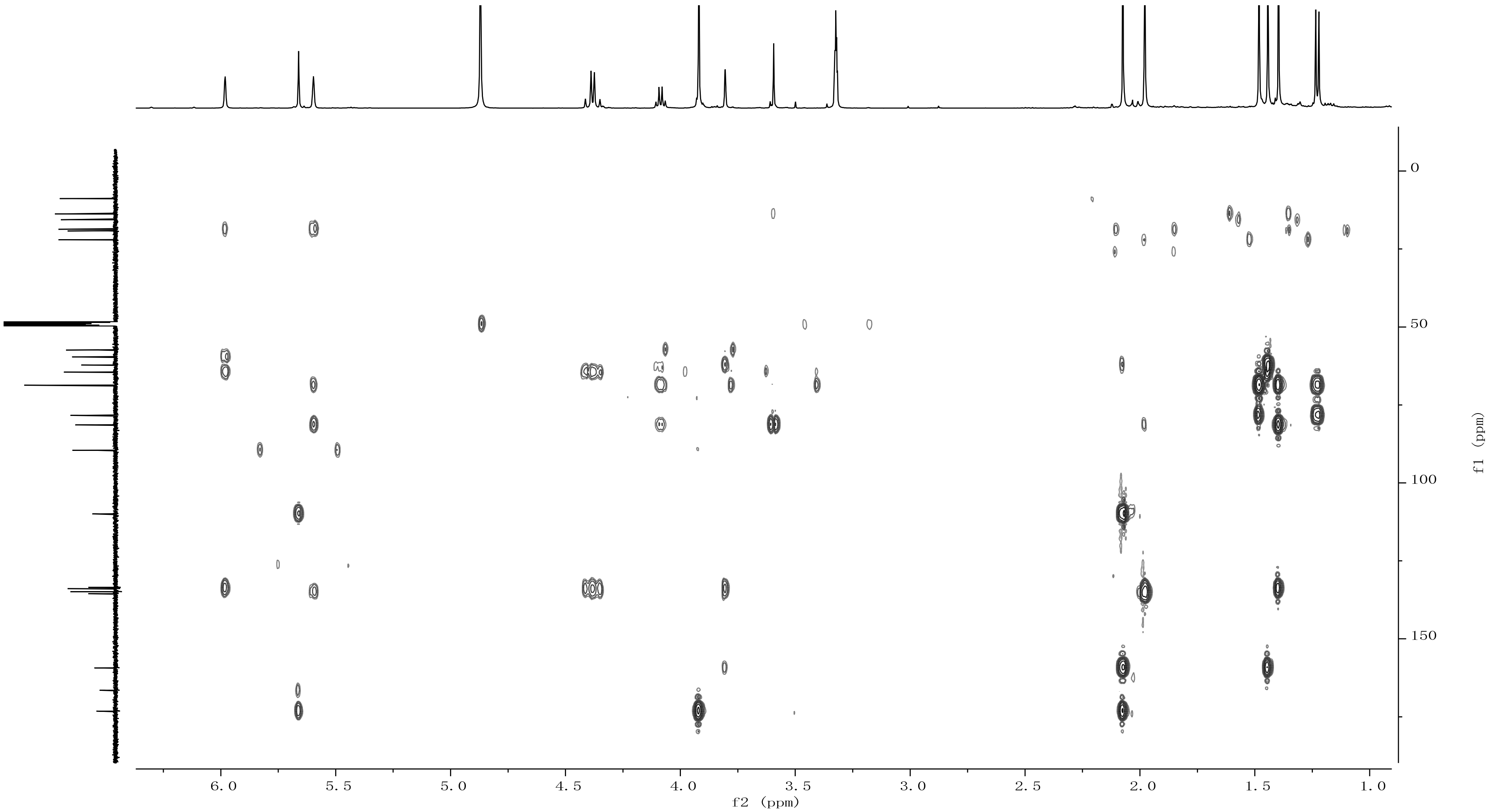

Figure S17. HMBC spectrum of penicicellarusin B (**4**) in CD_3OD

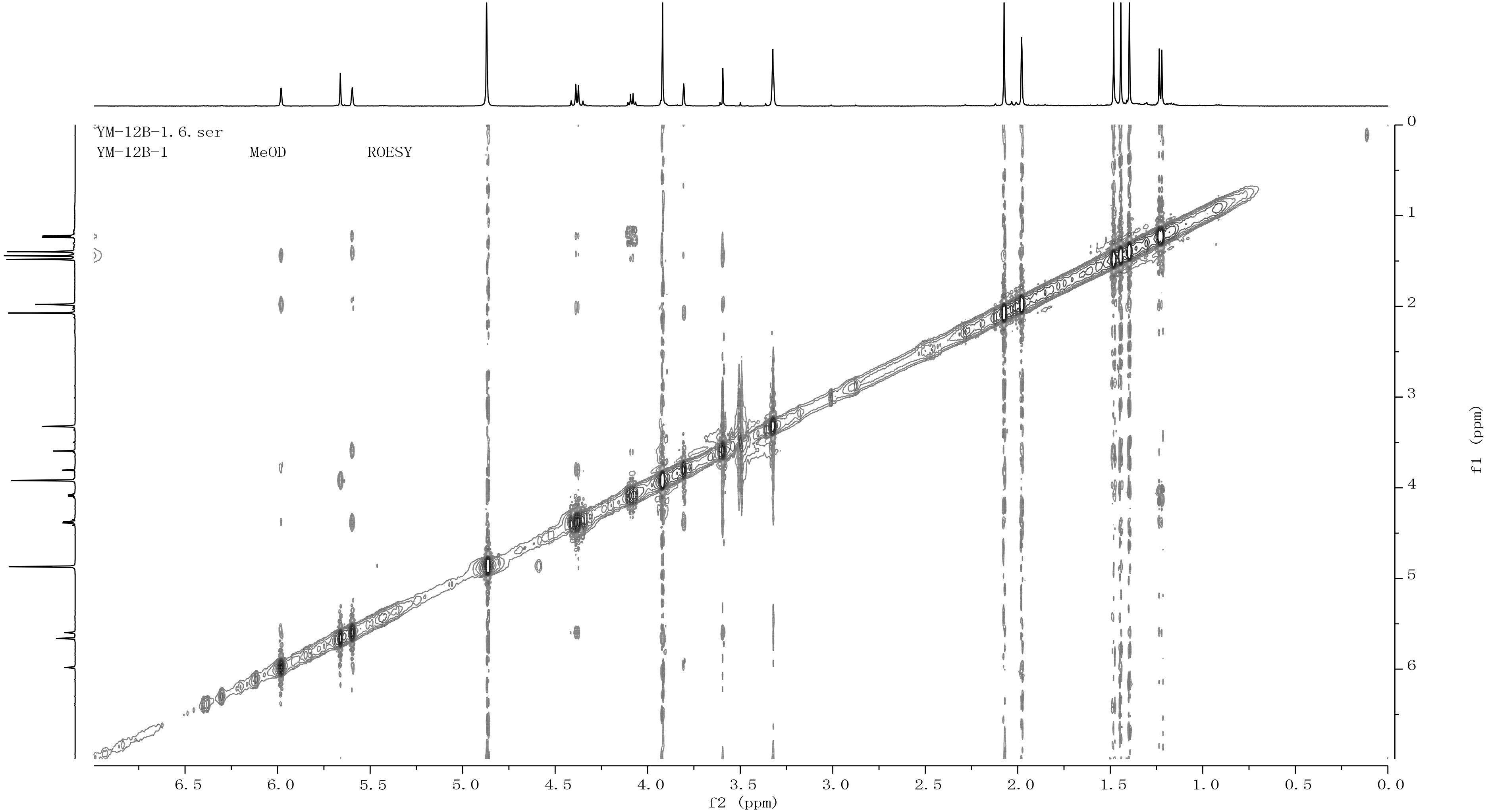

Figure S18. NOESY spectrum of penicicellarusin B (**4**) in CD_3OD

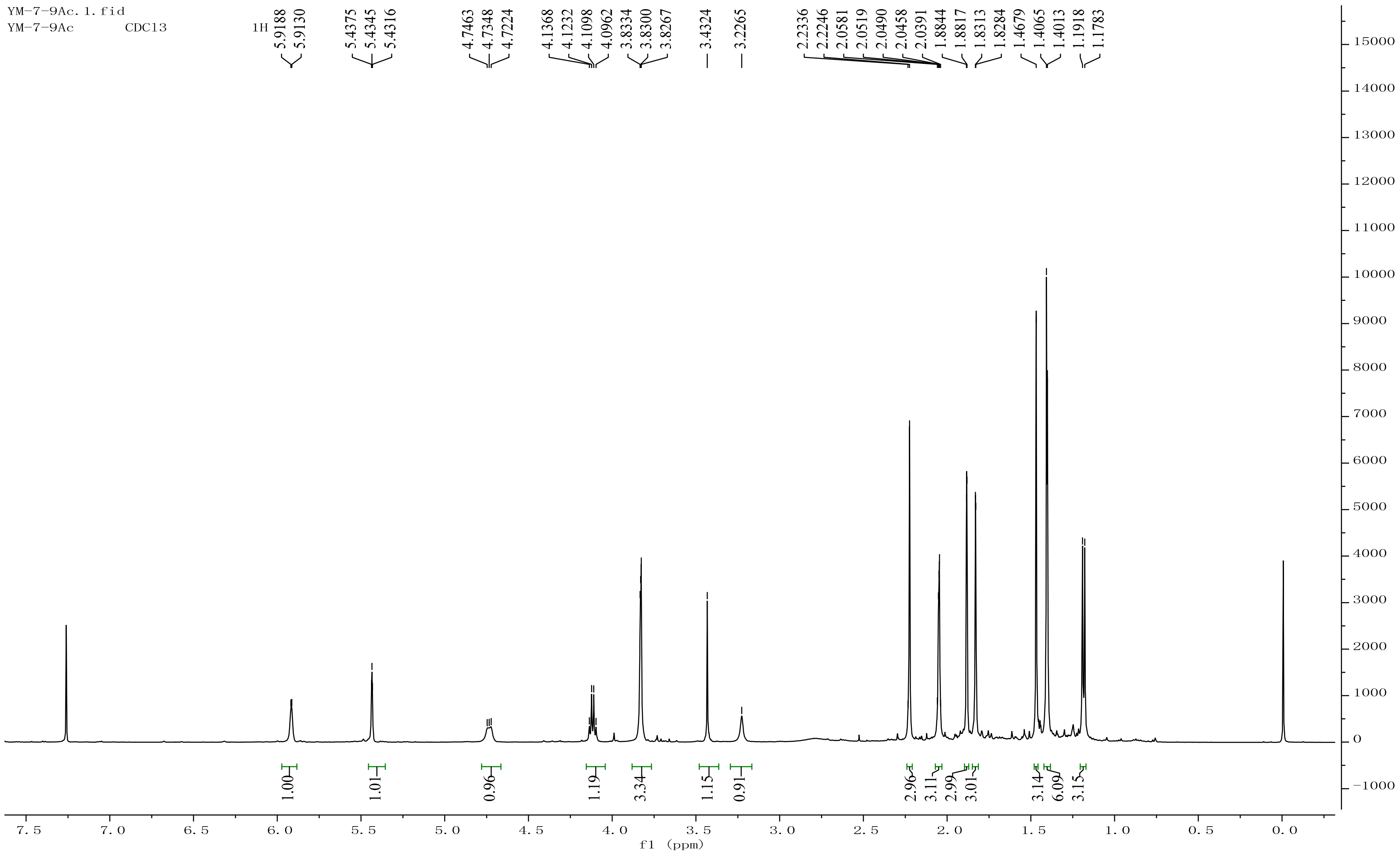

Figure S19. ^{1}H NMR spectrum of penicicellarusin C (**5**) in $CDCl_3$ (500 MHz)

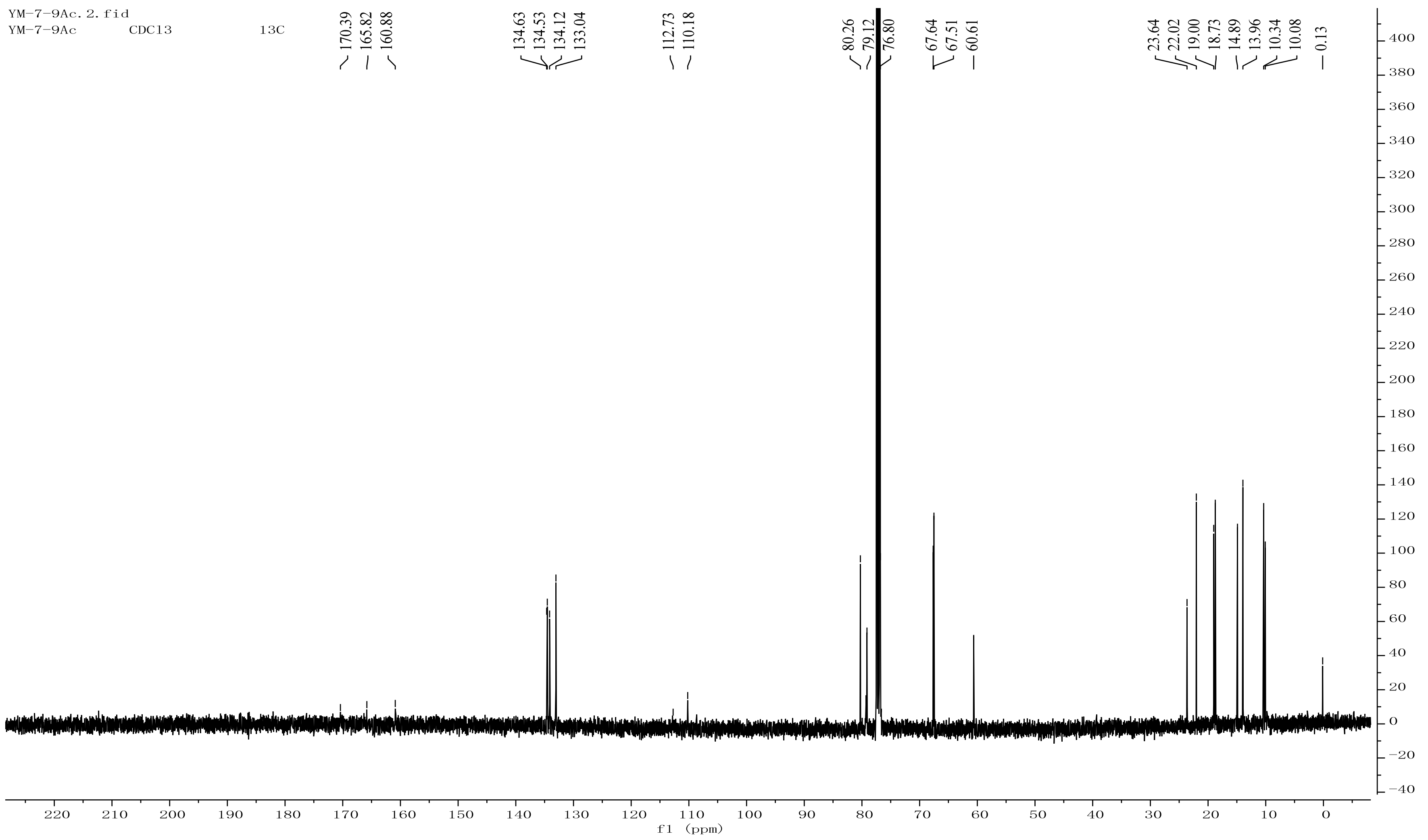

Figure S20. ^{13}C NMR spectrum of penicicellarusin C (**5**) in $CDCl_3$ (125 MHz)

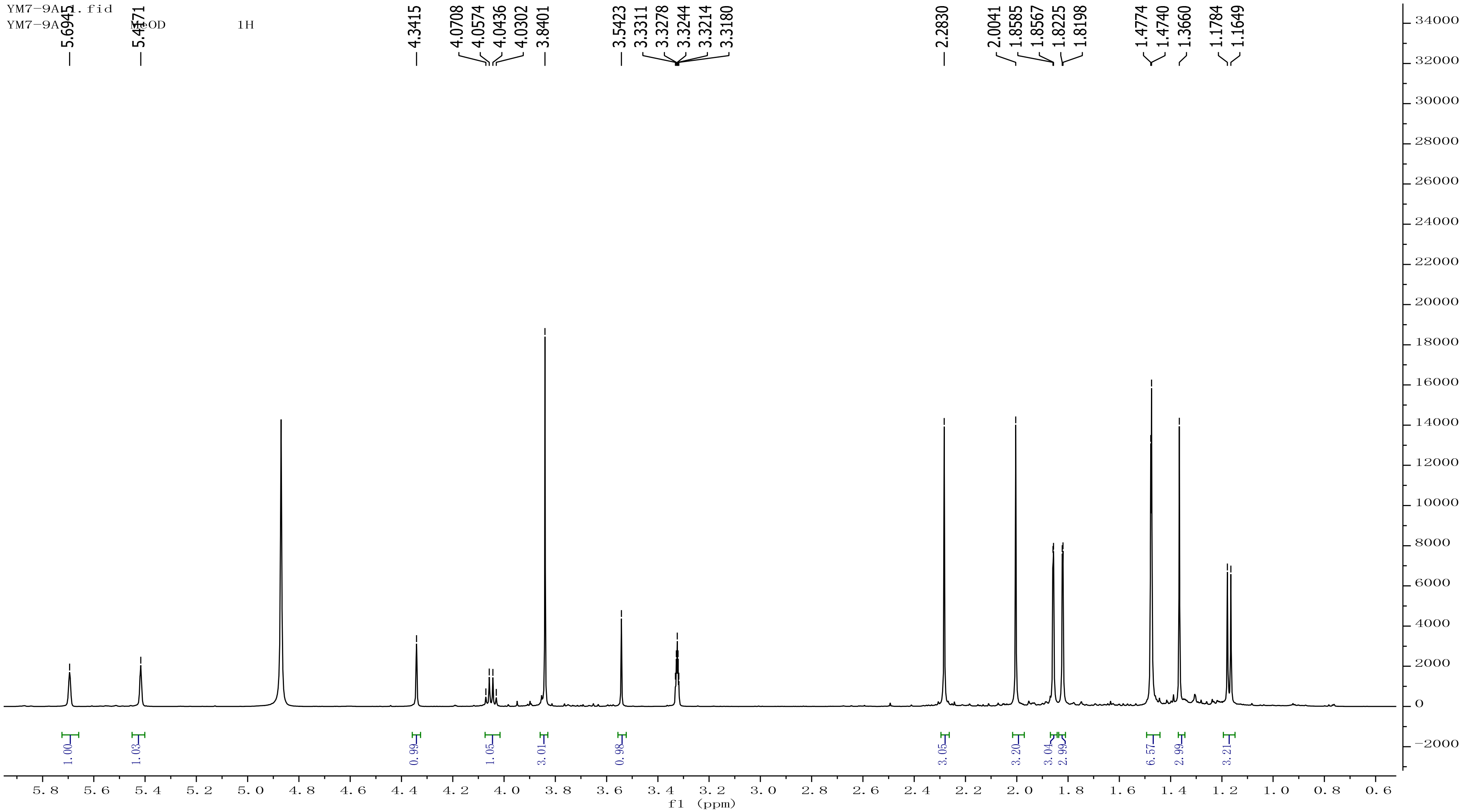

Figure S21. ^{1}H NMR spectrum of penicicellarusin C (**5**) in CD_3OD (500 MHz)

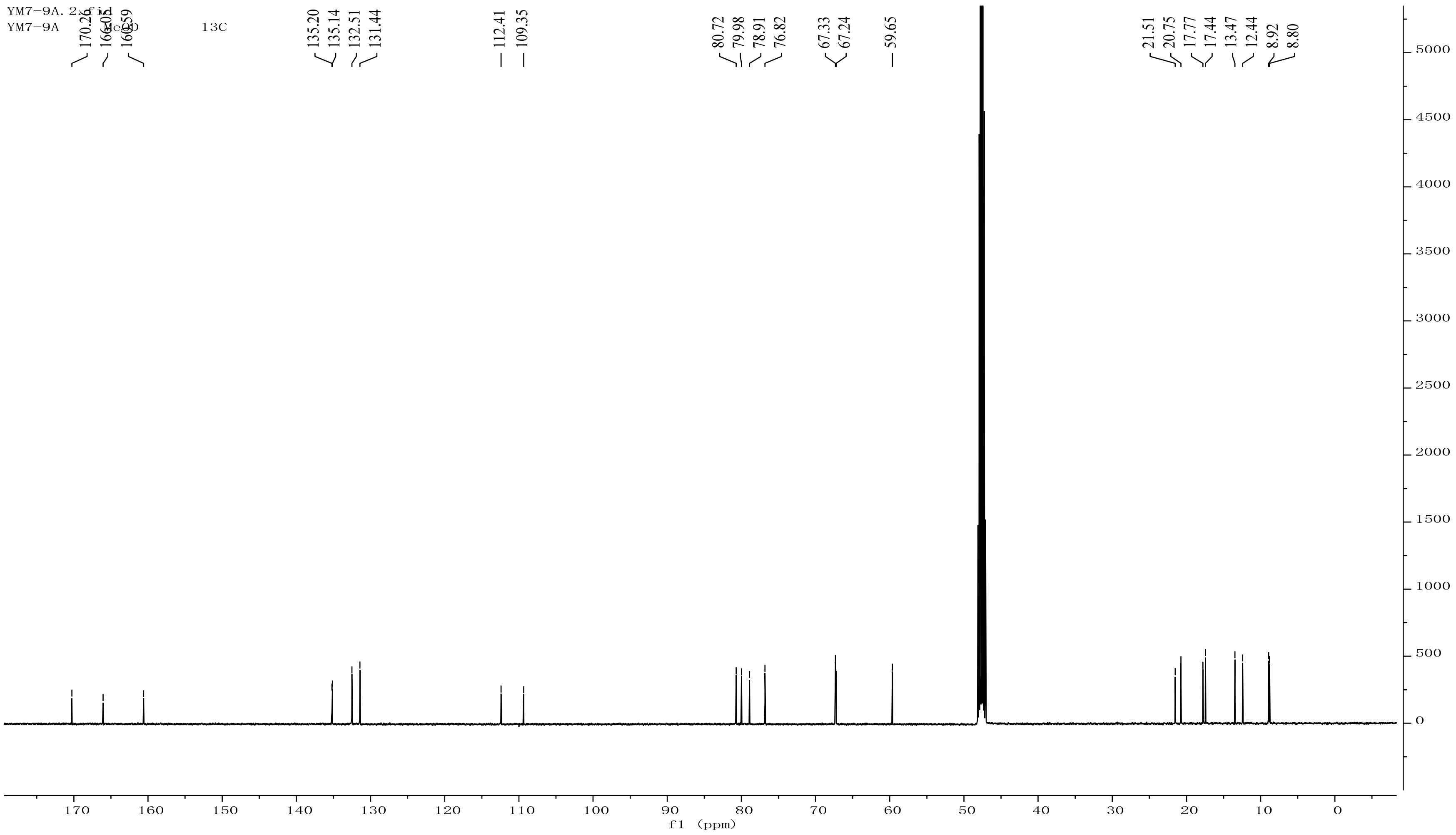

Figure S22. ^{13}C NMR spectrum of penicicellarusin C (**5**) in CD_3OD (125 MHz)

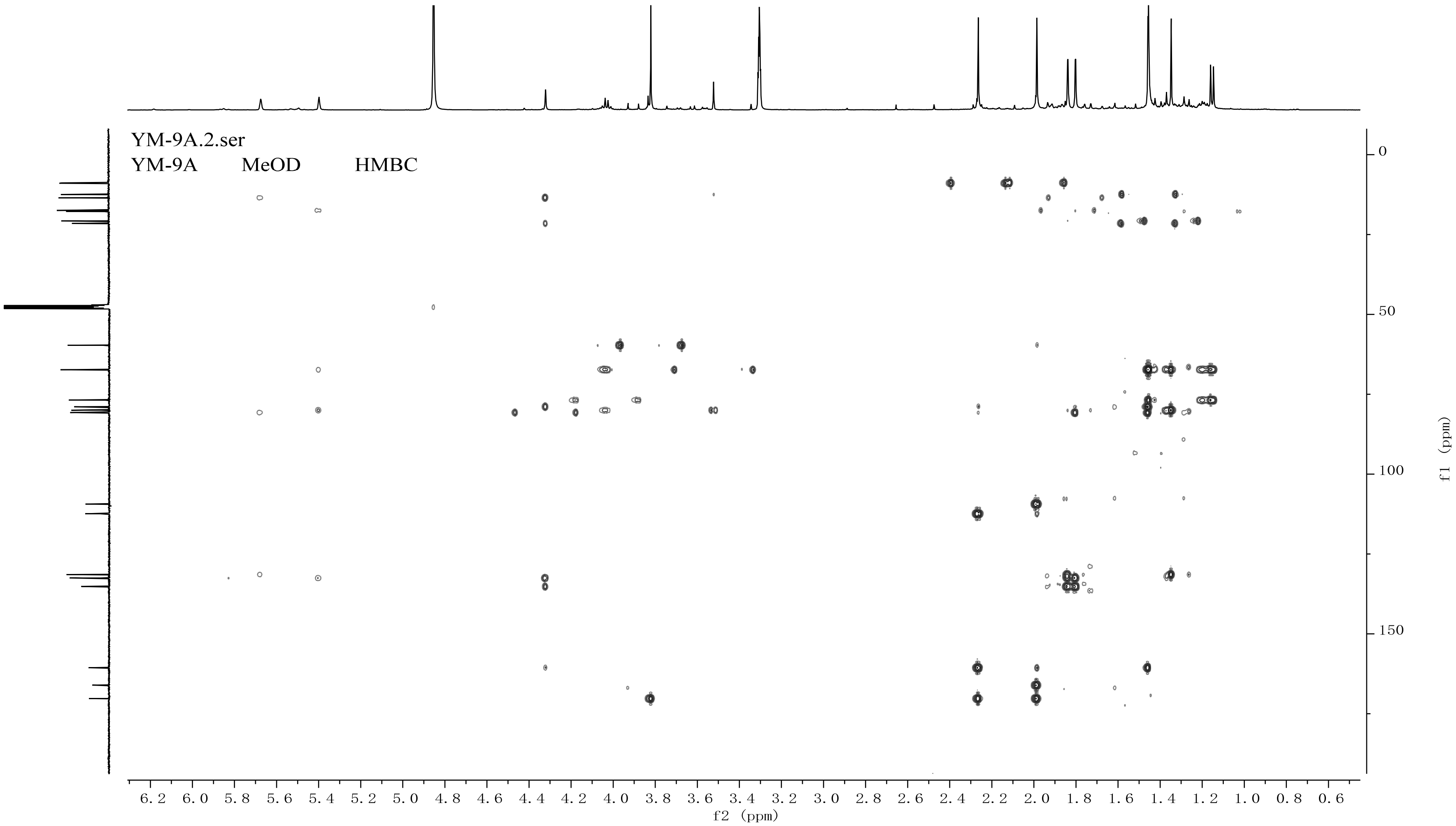

Figure S23. HMBC spectrum of penicicellarusin C (**5**) in CD_3OD

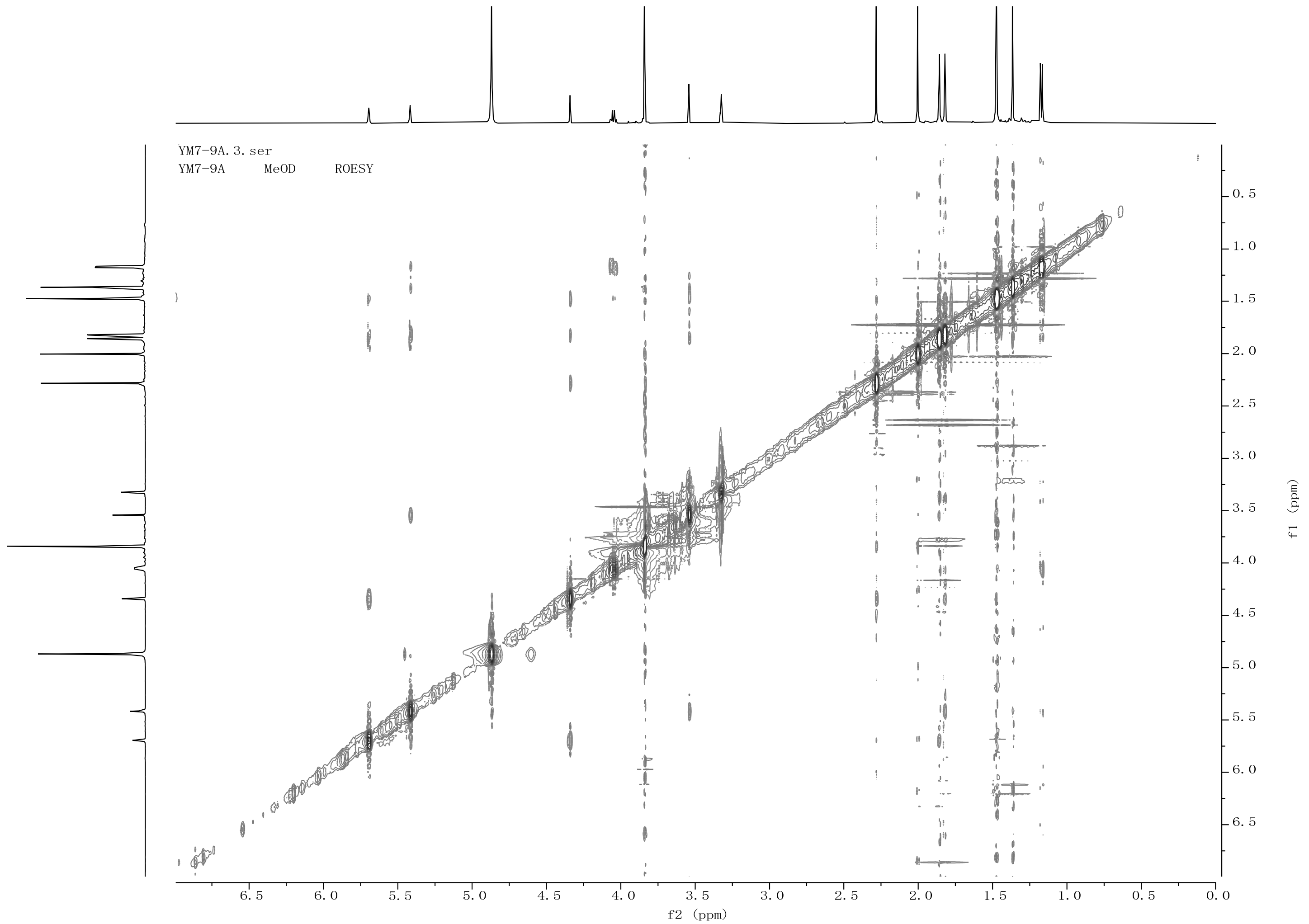

Figure S24. NOESY spectrum of penicicellarusin C (**5**) in CD_3OD

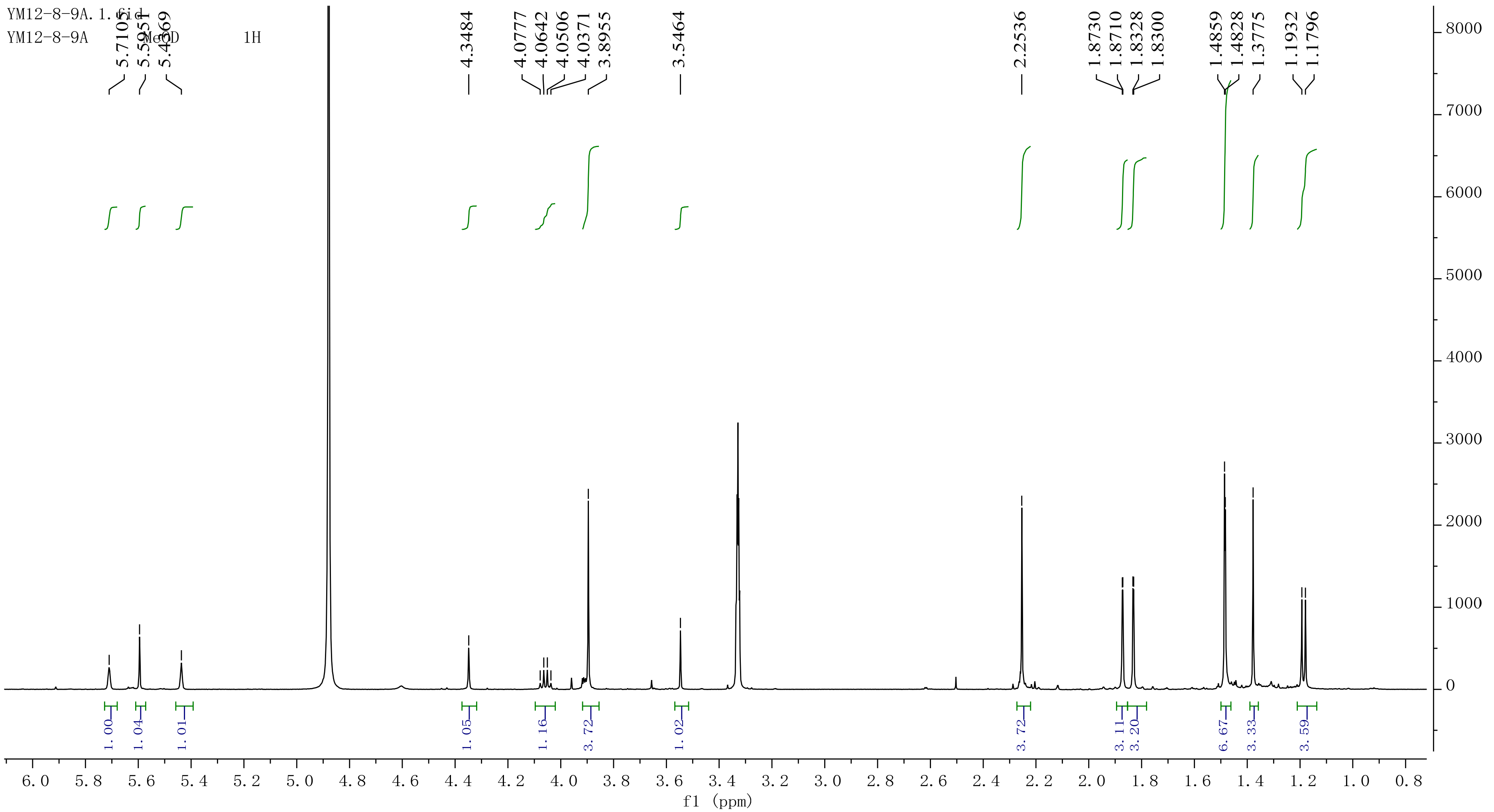

Figure S25. ^{1}H NMR spectrum of penicicellarusin D (**6**) in CD_3OD (500 MHz)

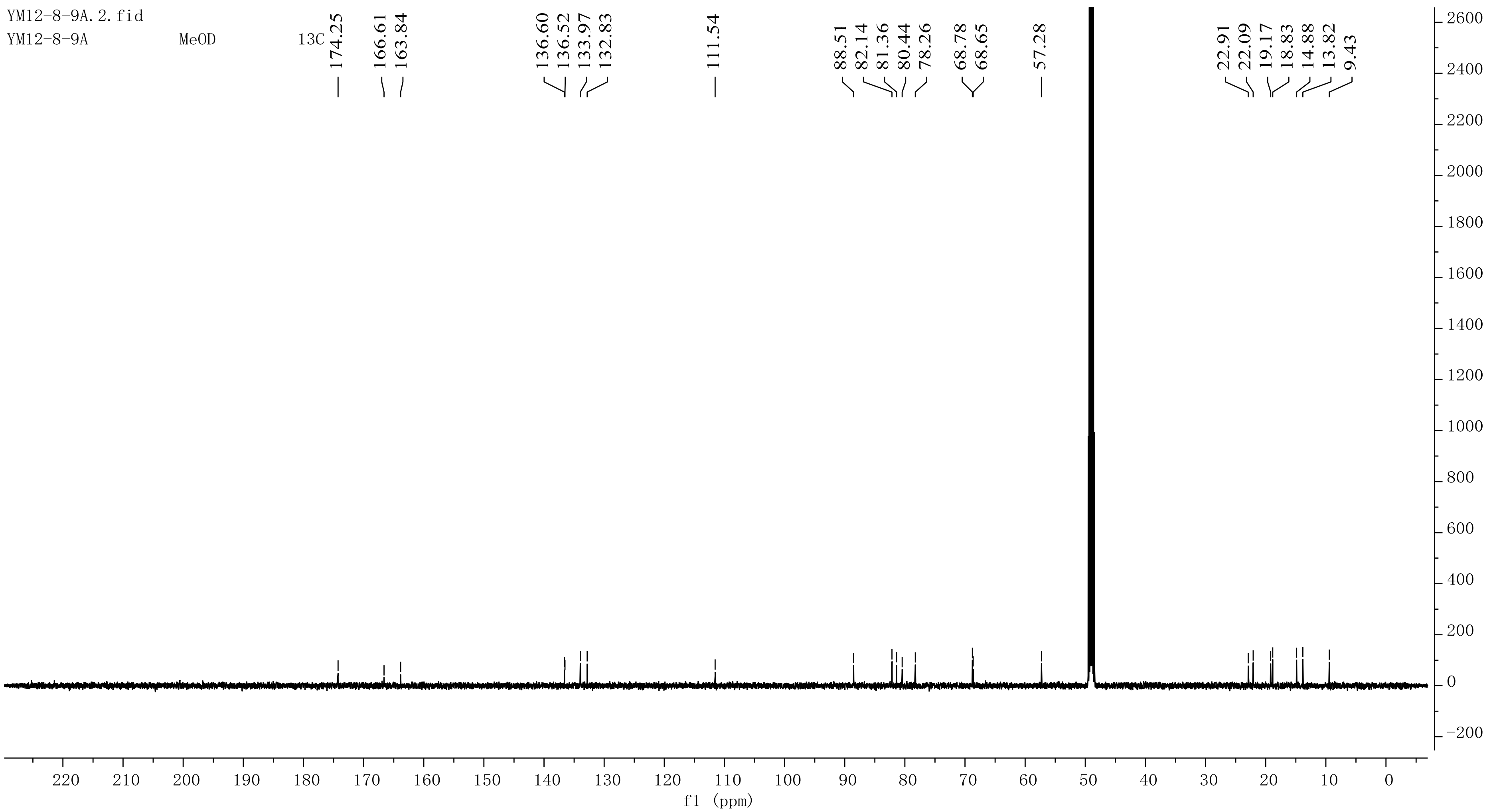

Figure S26. ^{13}C NMR spectrum of penicicellarusin D (**6**) in CD_3OD (125 MHz)

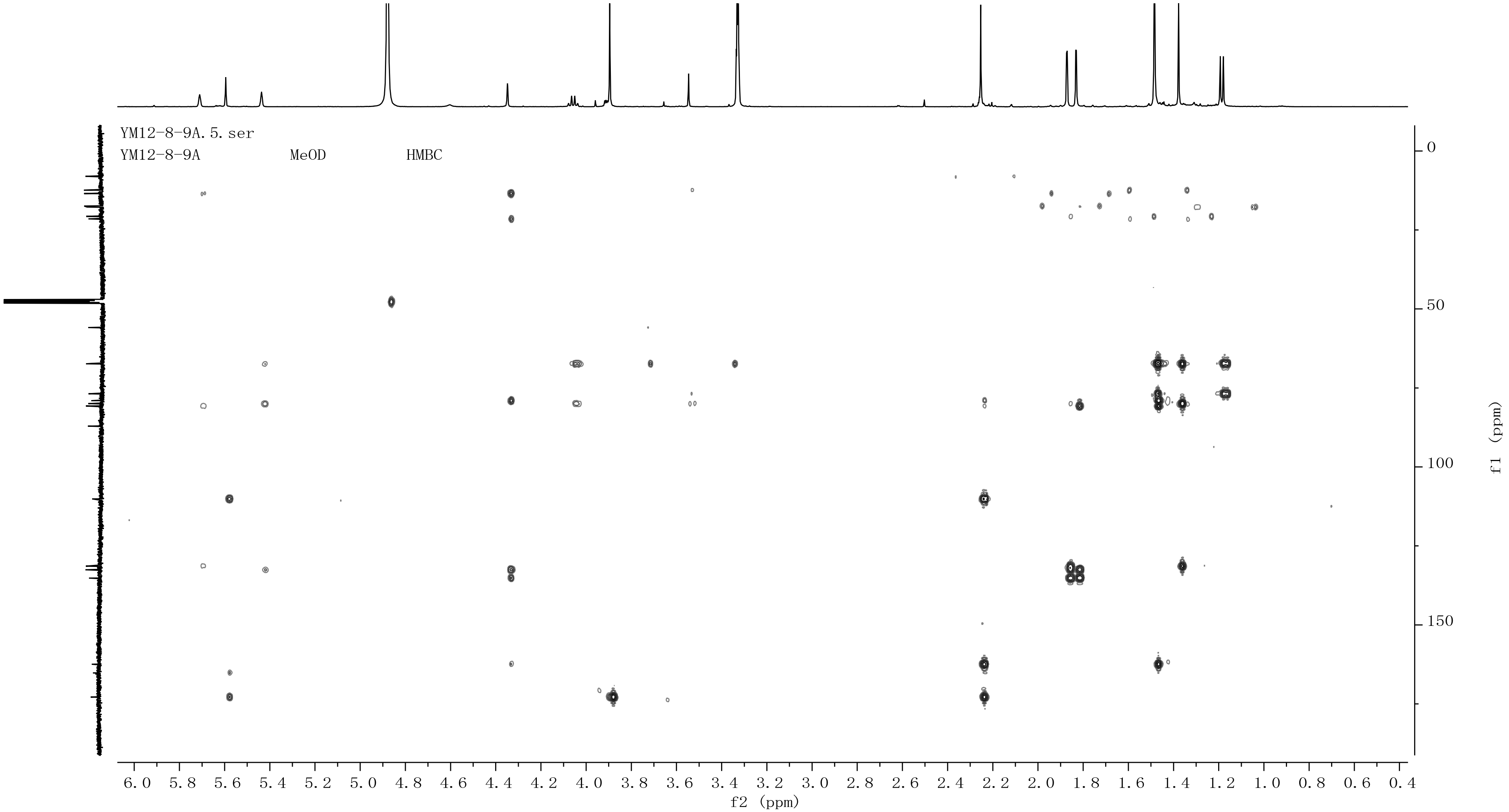

Figure S27. HMBC spectrum of penicicellarusin D (**6**) in CD_3OD

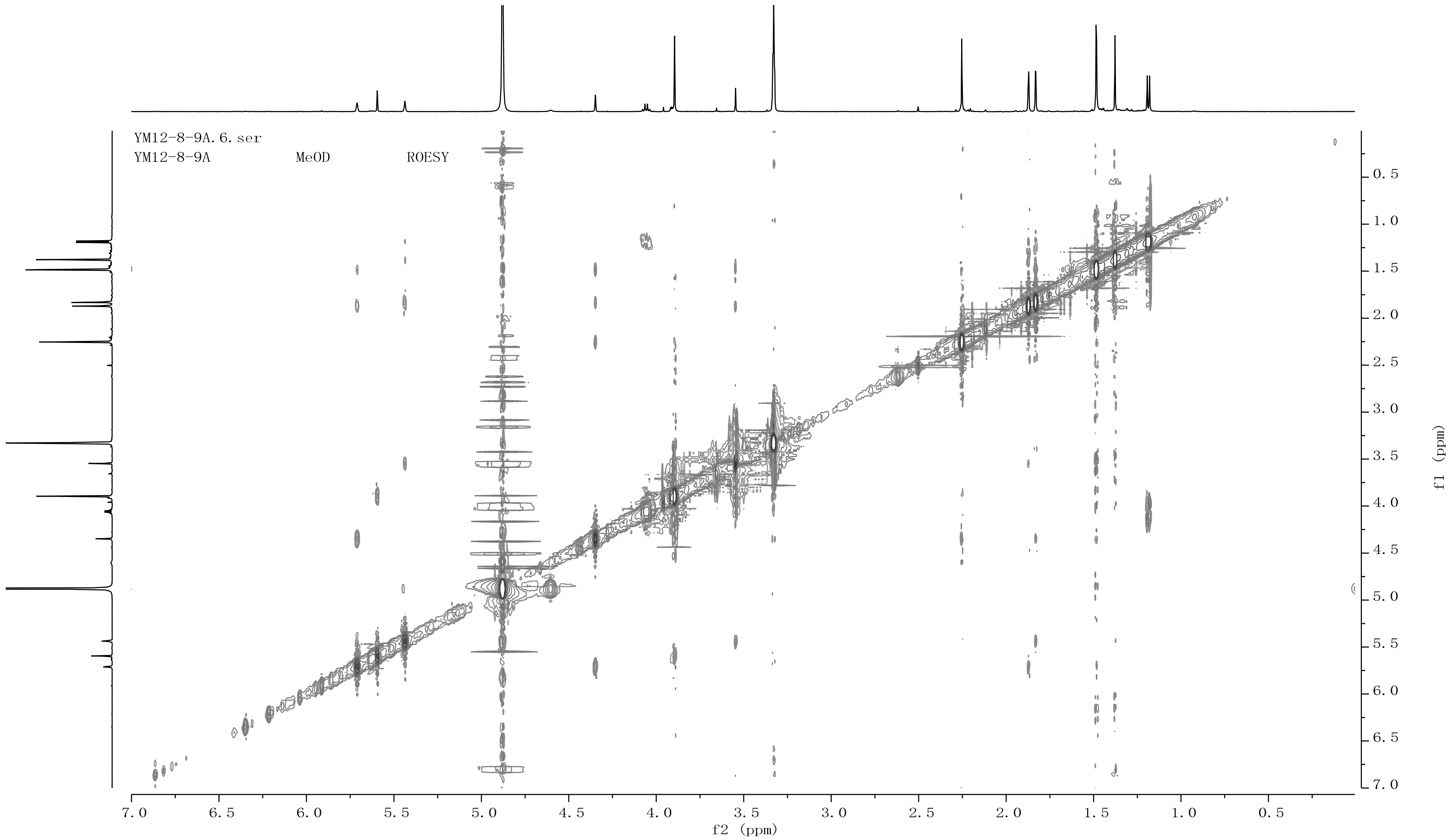

Figure S28. NOESY spectrum of penicicellarusin D (**6**) in CD_3OD

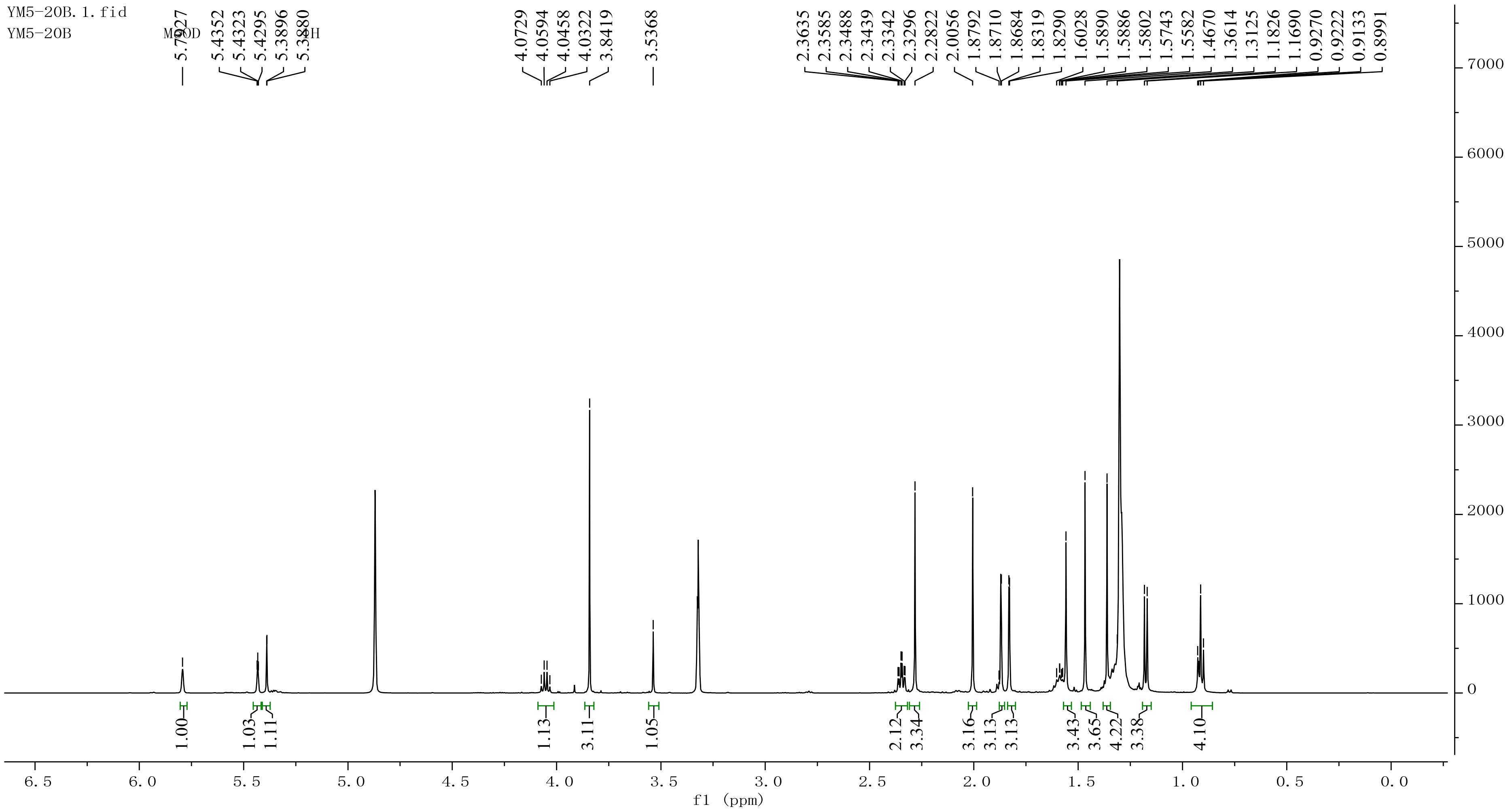

Figure S29. ^{1}H NMR spectrum of penicicellarusin E (**7**) in CD_3OD (500 MHz)

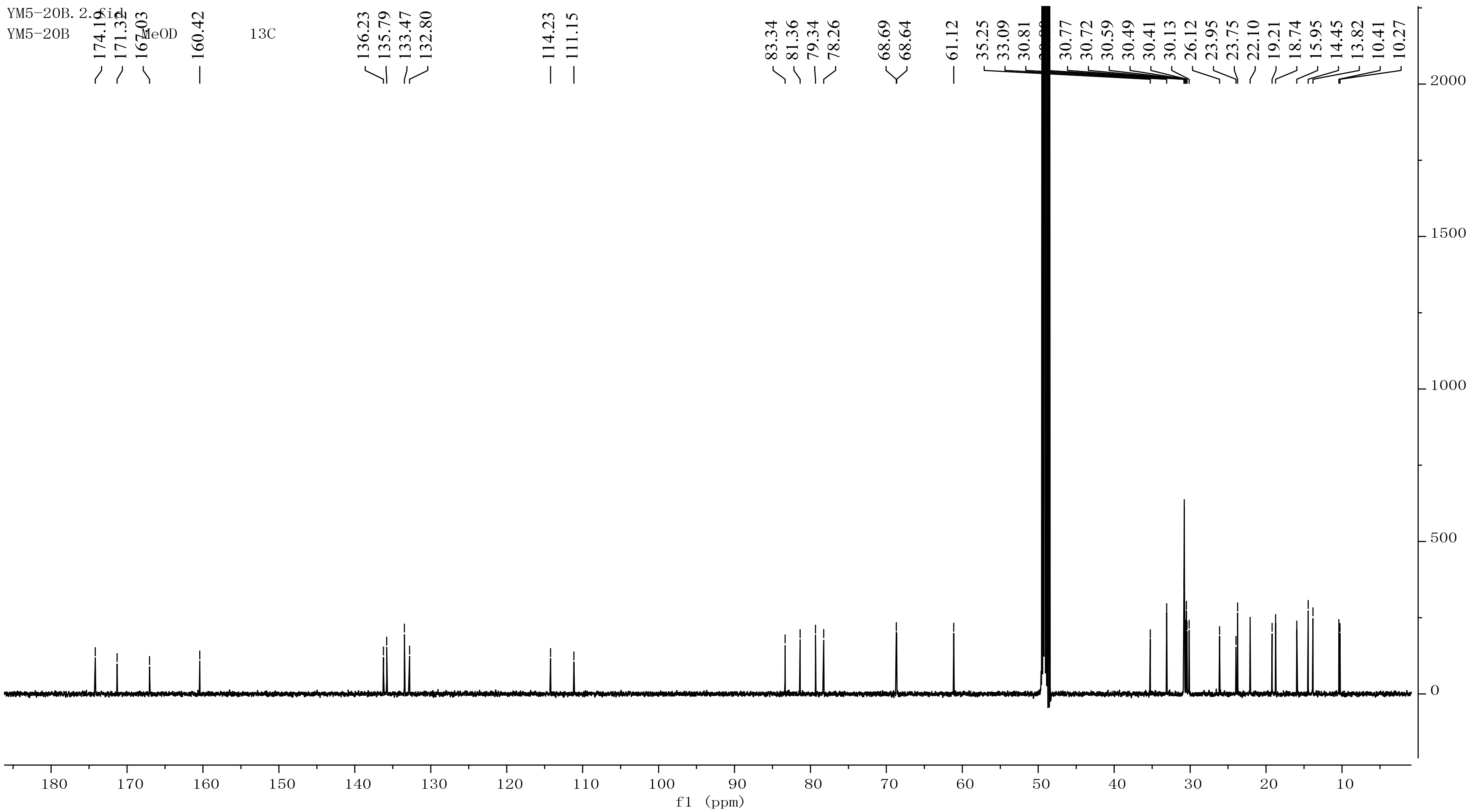

Figure S30. ^{13}C NMR spectrum of penicicellarusin E (**7**) in CD_3OD (125 MHz)

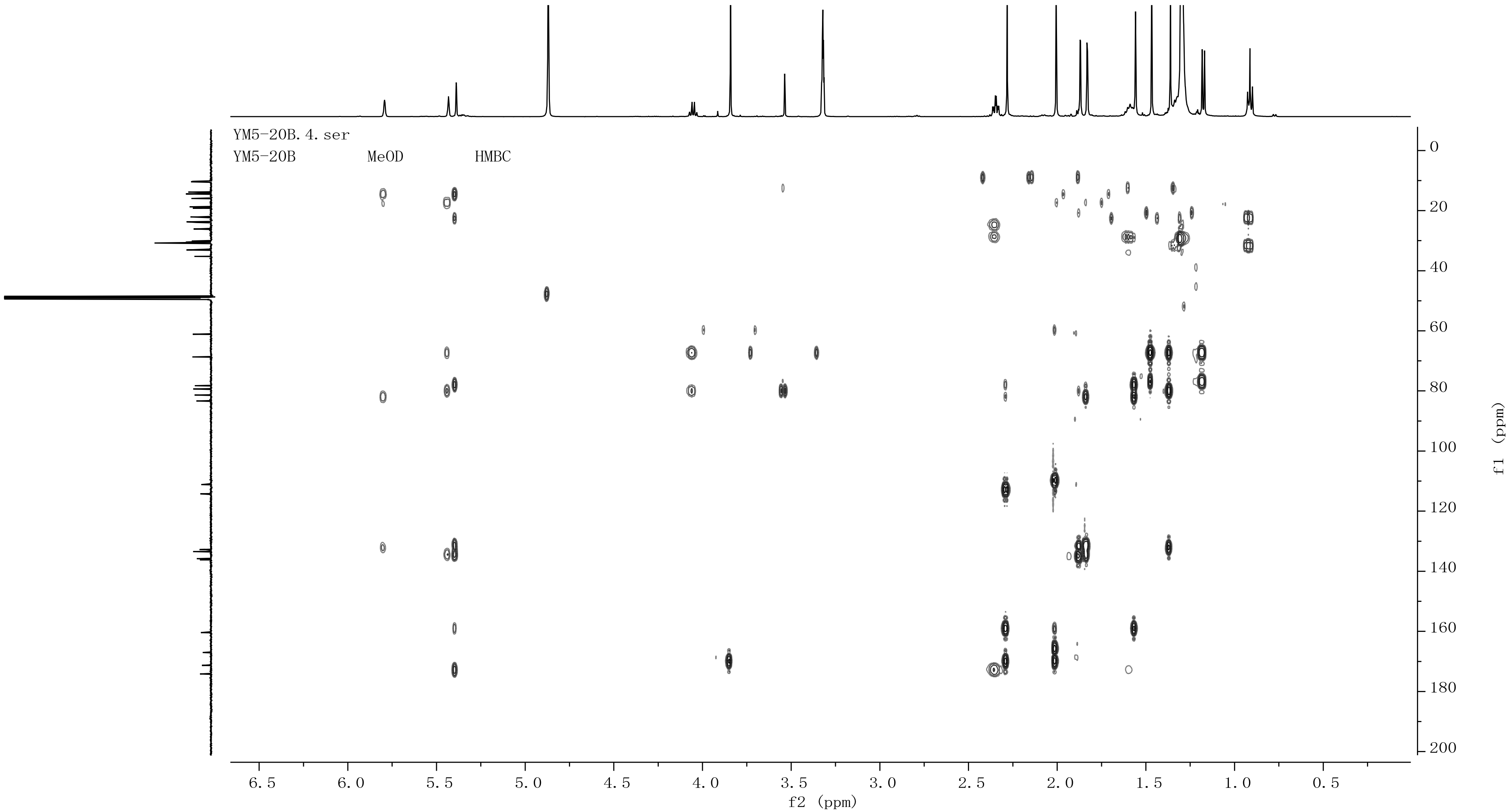

Figure S31. HMBC spectrum of penicicellarusin E (**7**) in CD_3OD

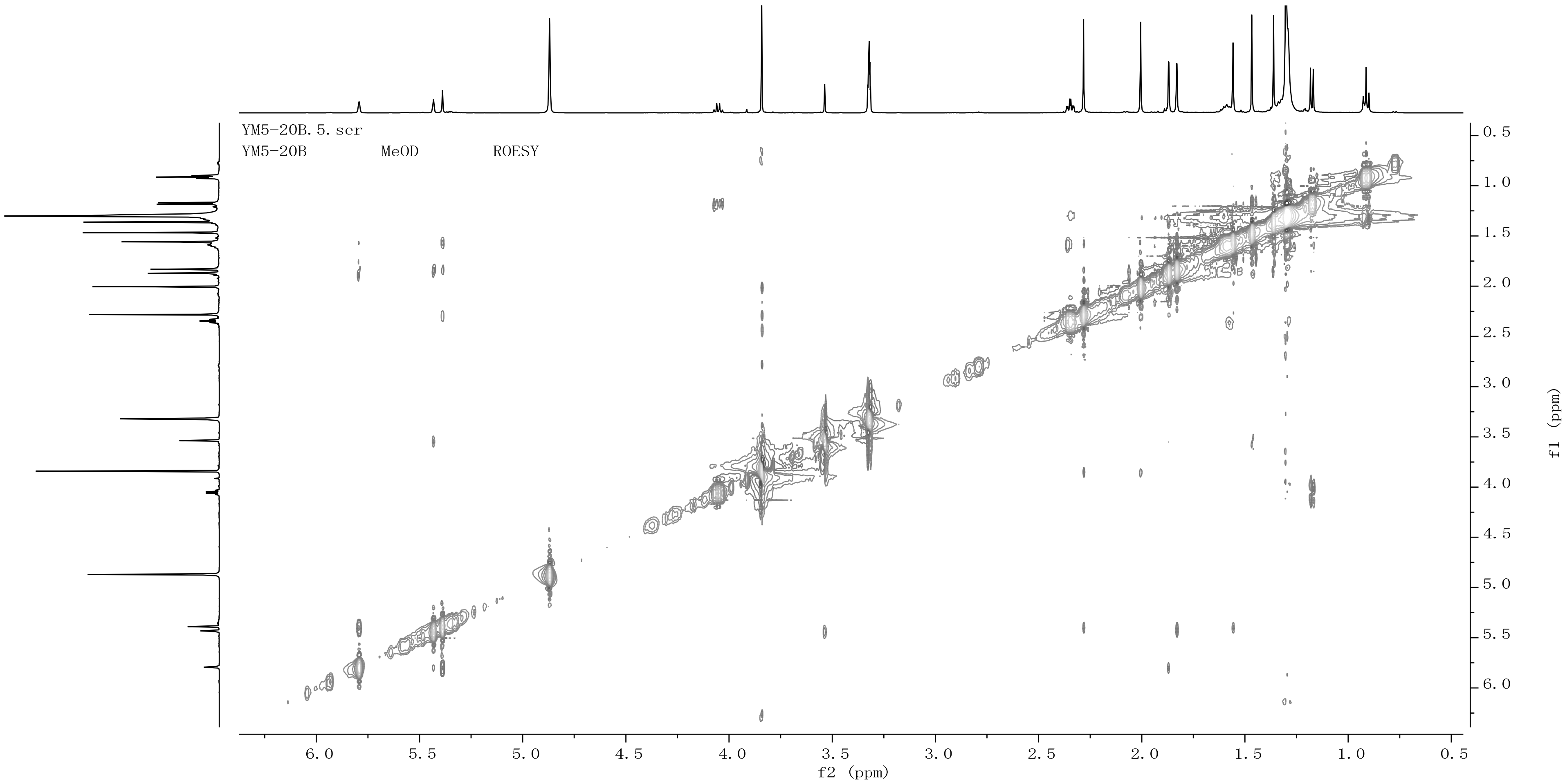

Figure S32. NOESY spectrum of penicicellarusin E (**7**) in CD_3OD

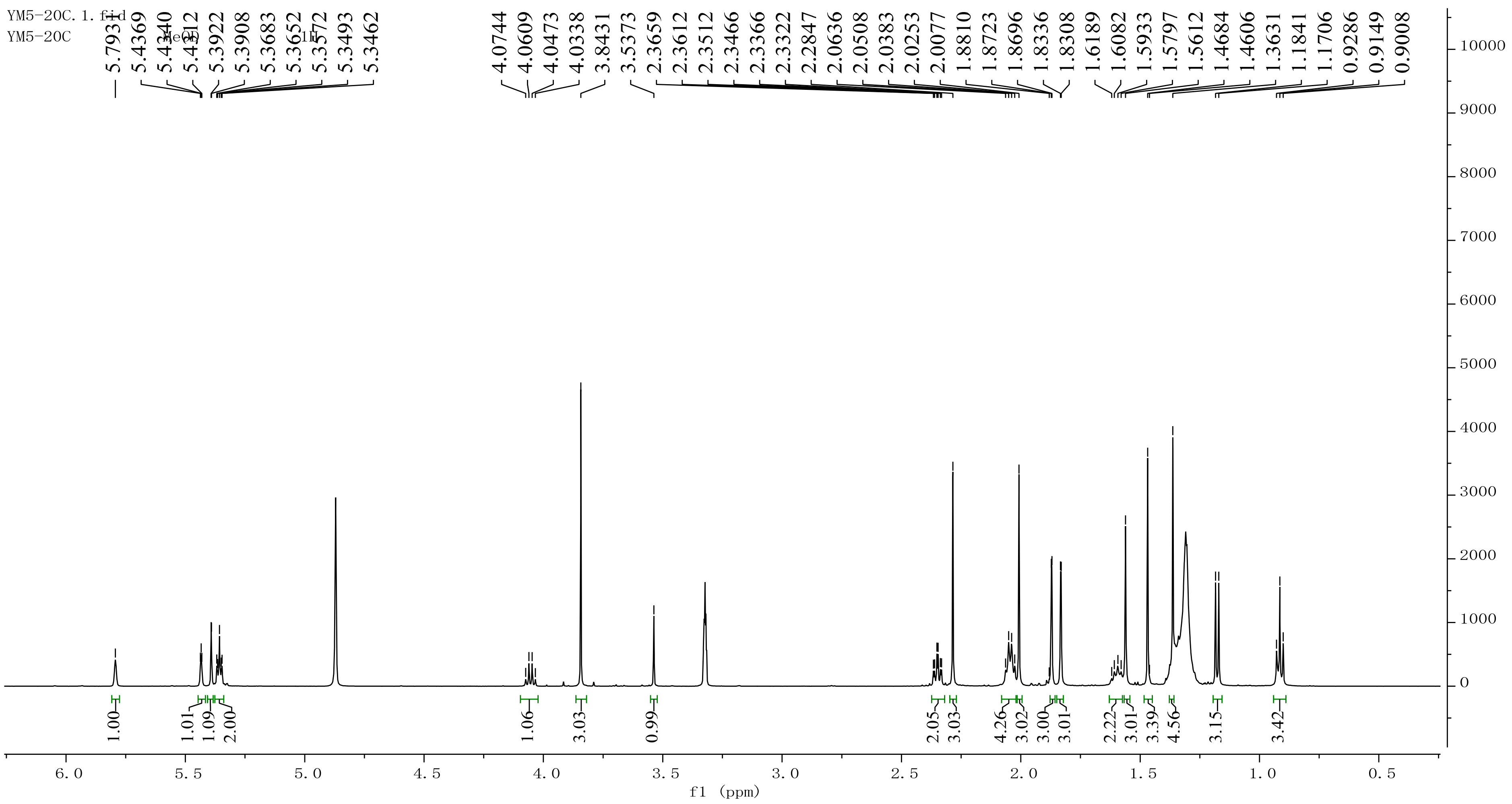

Figure S33. ^{1}H NMR spectrum of penicicellarusin F (**8**) in CD_3OD (500 MHz)

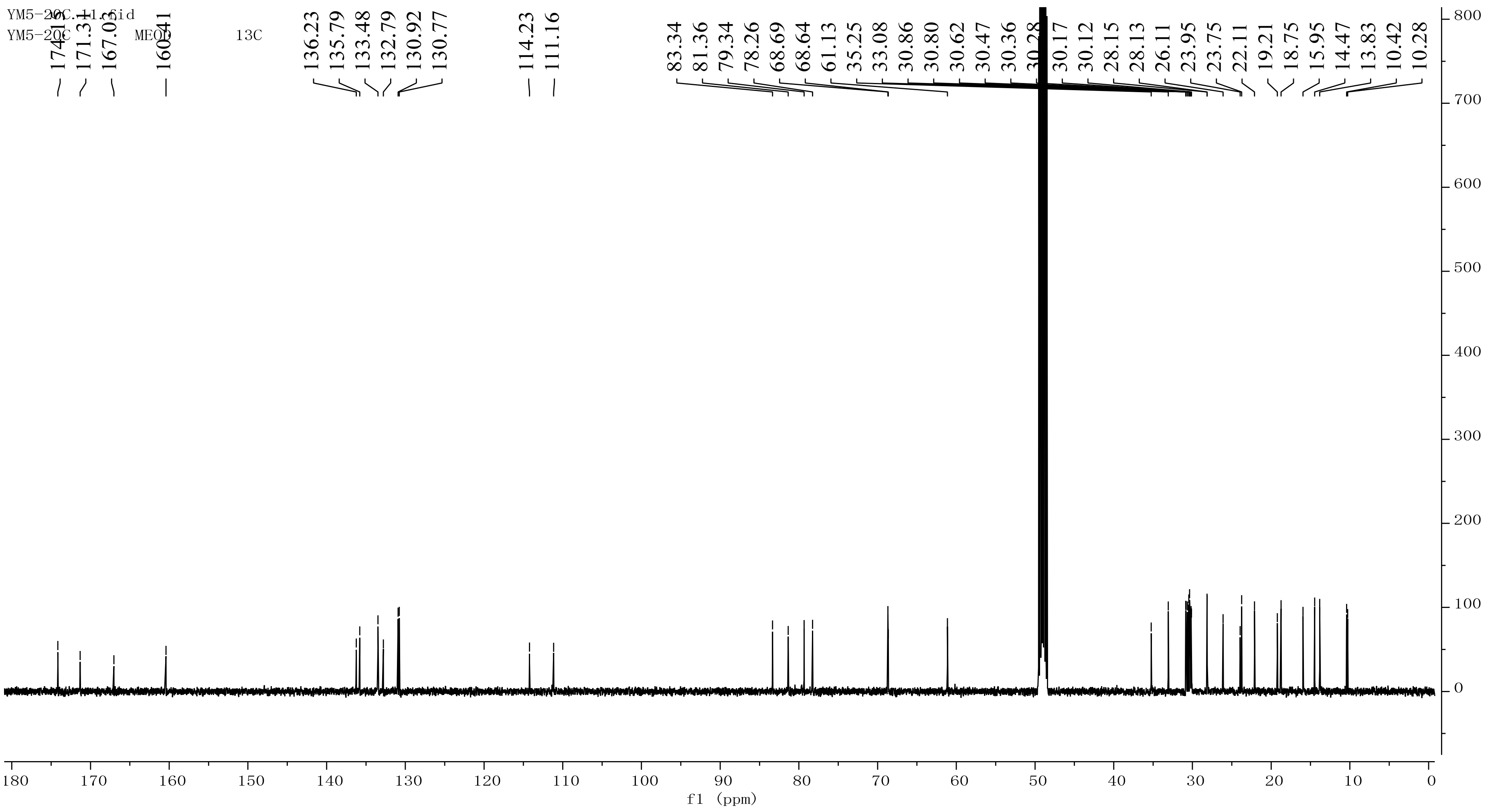

Figure S34. ^{13}C NMR spectrum of penicicellarusin F (**8**) in CD_3OD (125 MHz)

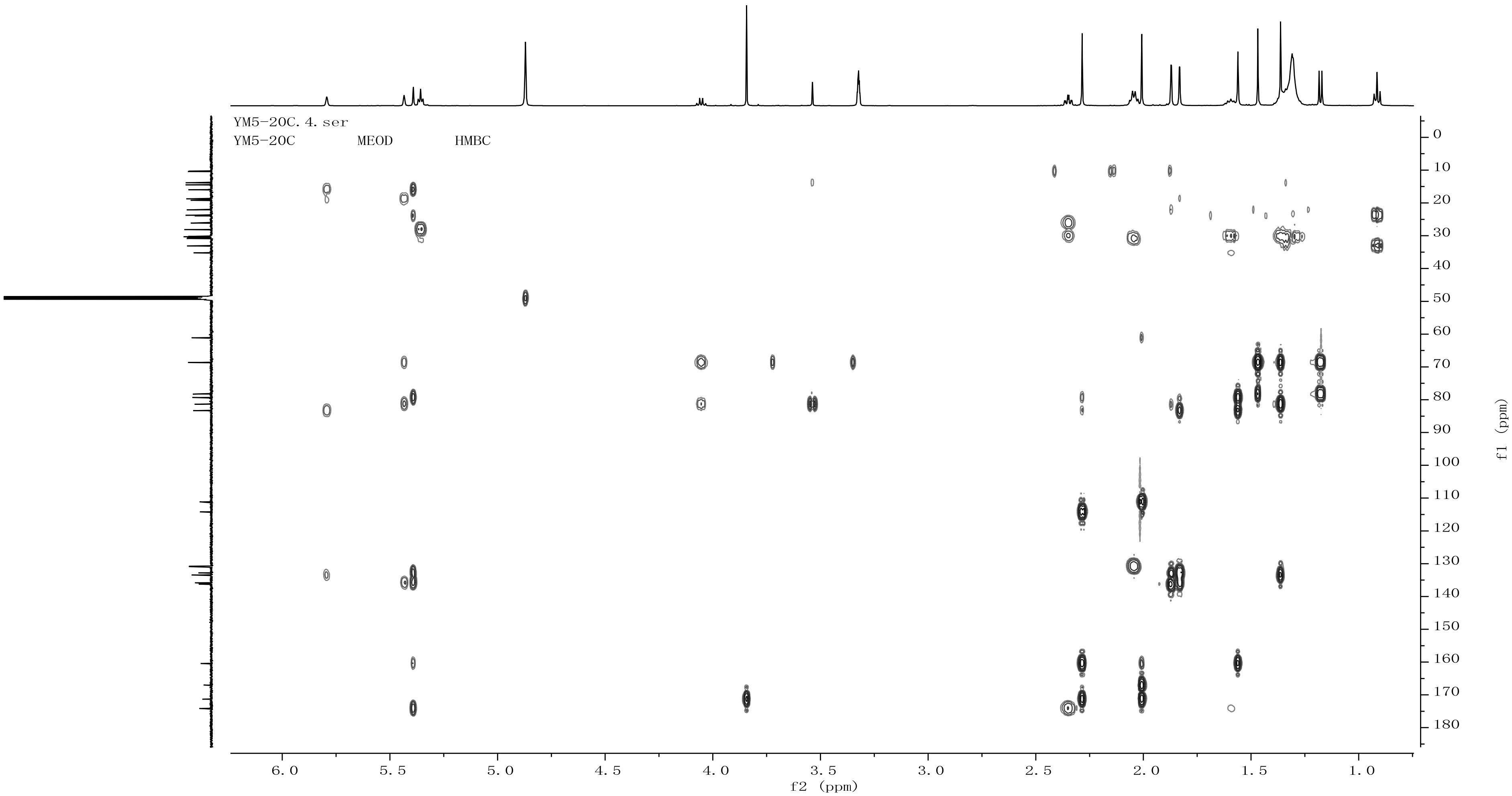

Figure S35. HMBC spectrum of penicicellarusin F (**8**) in CD_3OD

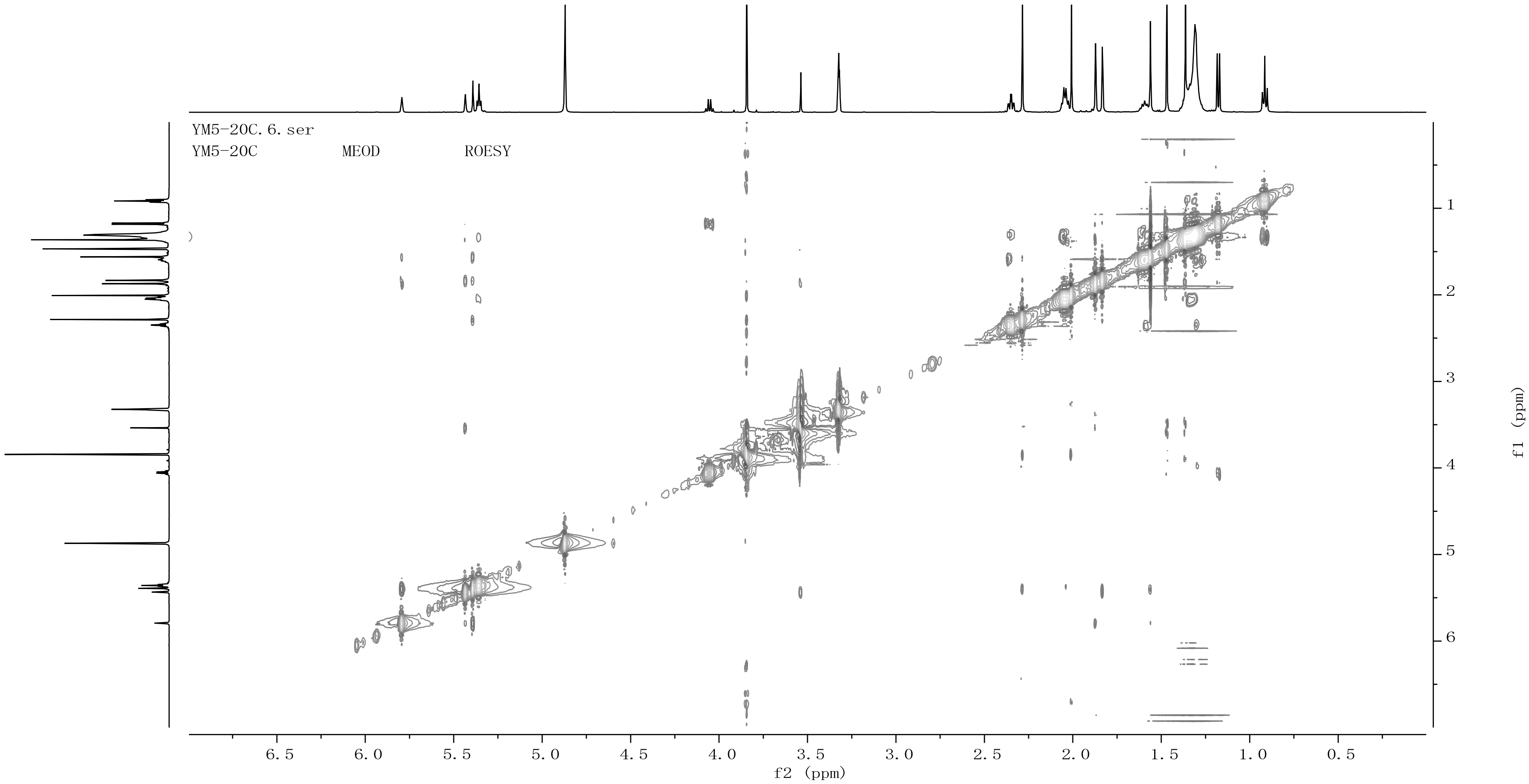

Figure S36. NOESY spectrum of penicicellarusin F (**8**) in CD_3OD

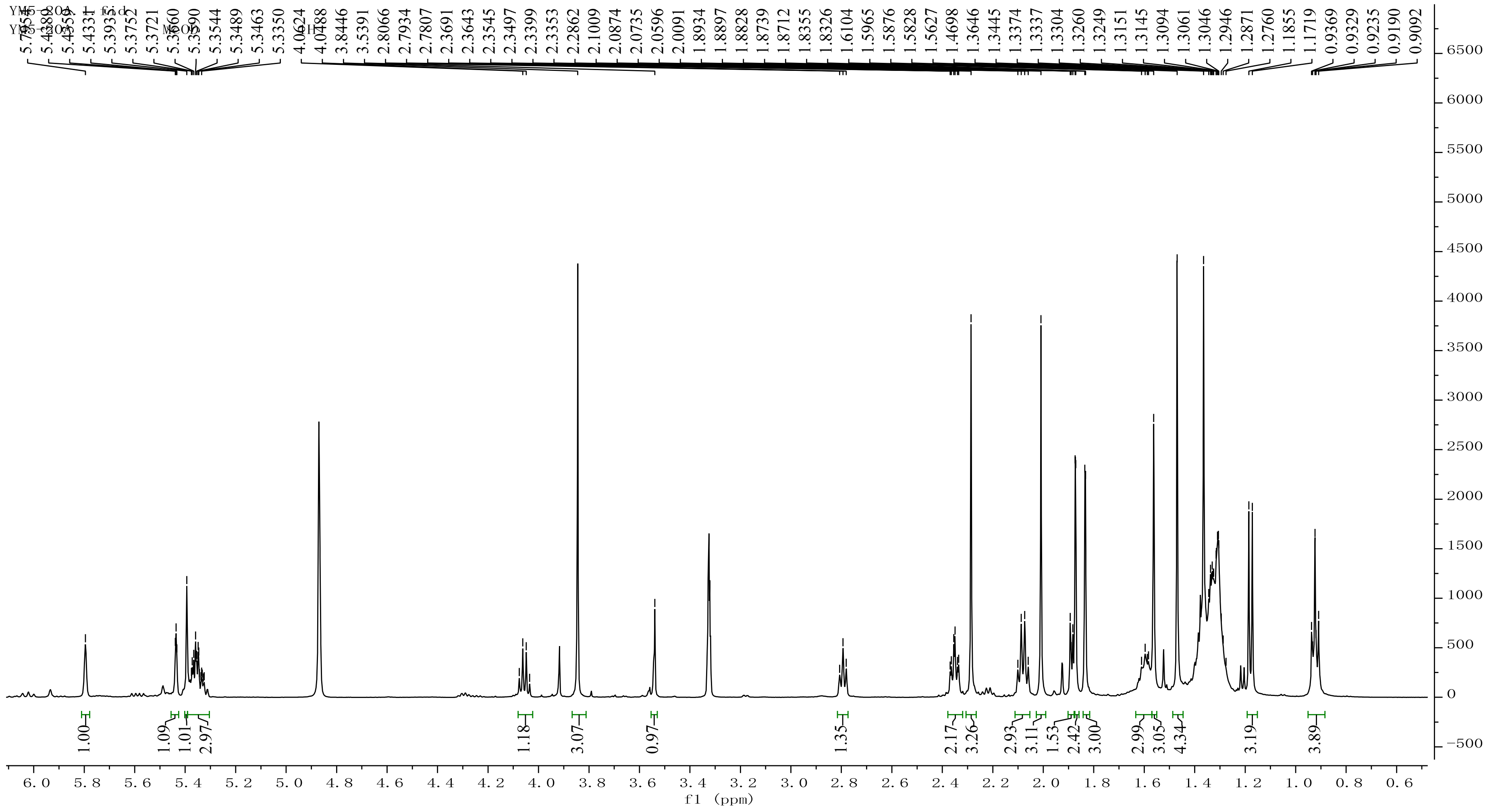

Figure S37. ^{1}H NMR spectrum of penicicellarusin G (**9**) in CD_3OD (500 MHz)

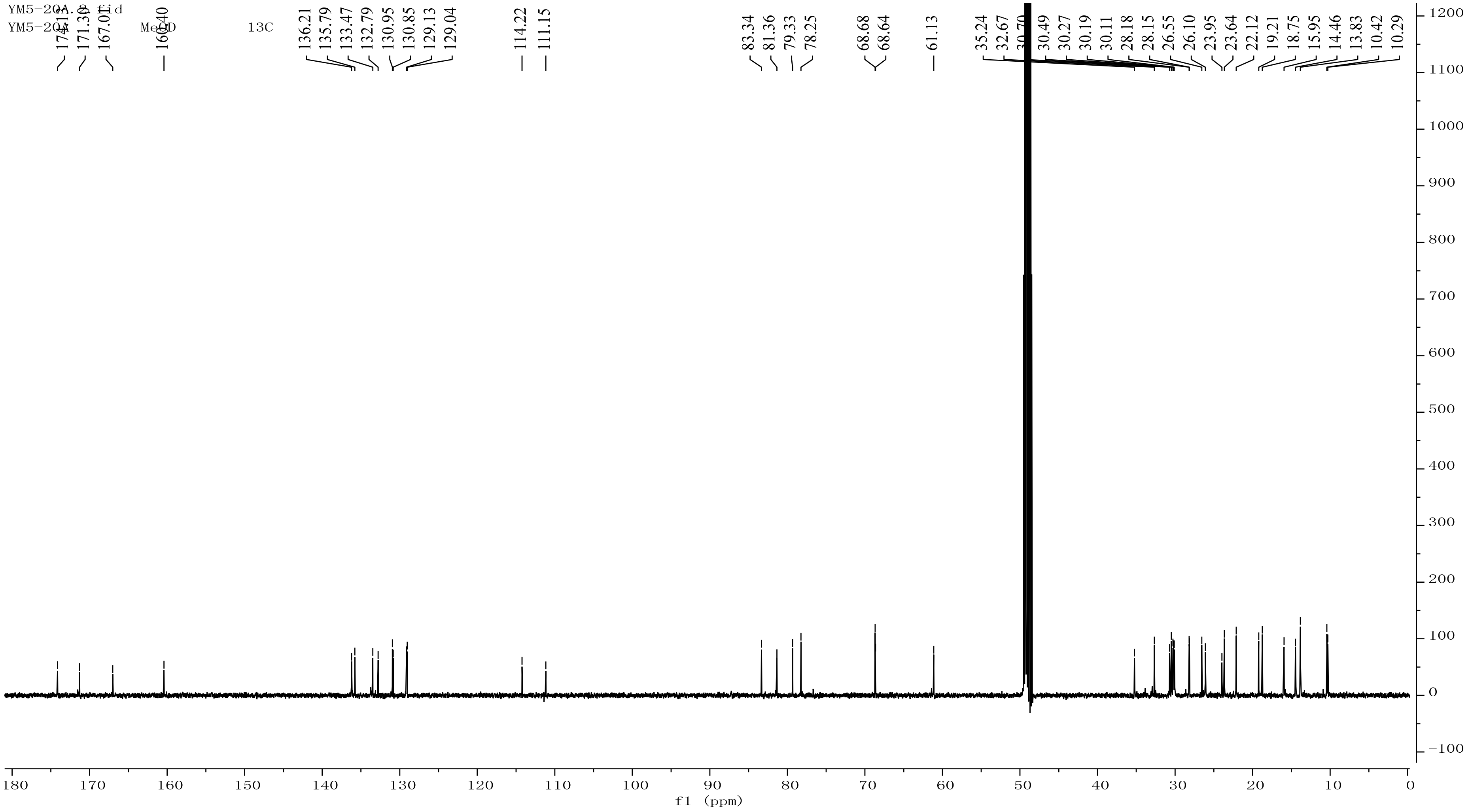

Figure S38. ^{13}C NMR spectrum of penicicellarusin G (**9**) in CD_3OD (125 MHz)

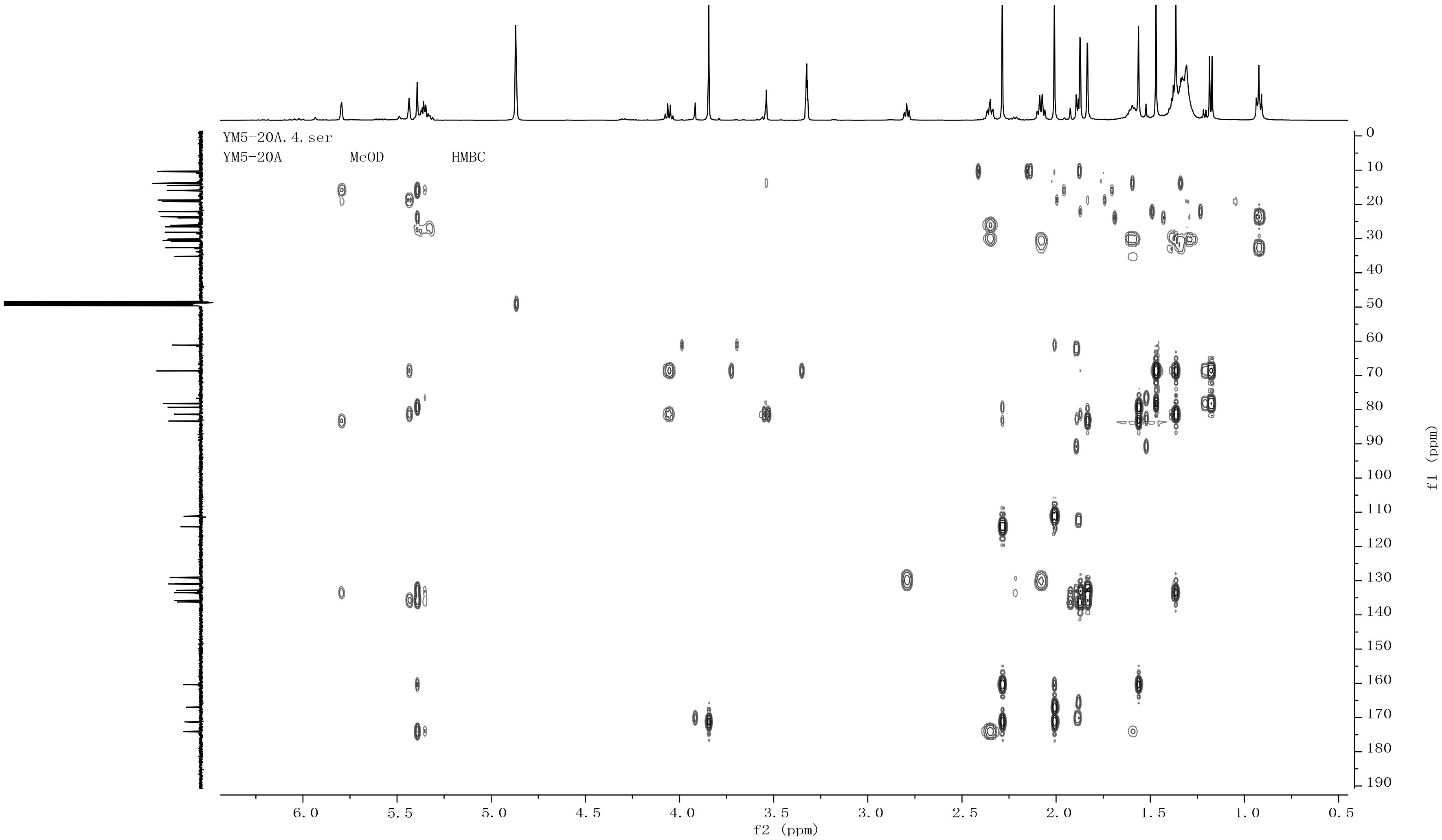

Figure S39. HMBC spectrum of penicicellarusin G (**9**) in CD_3OD

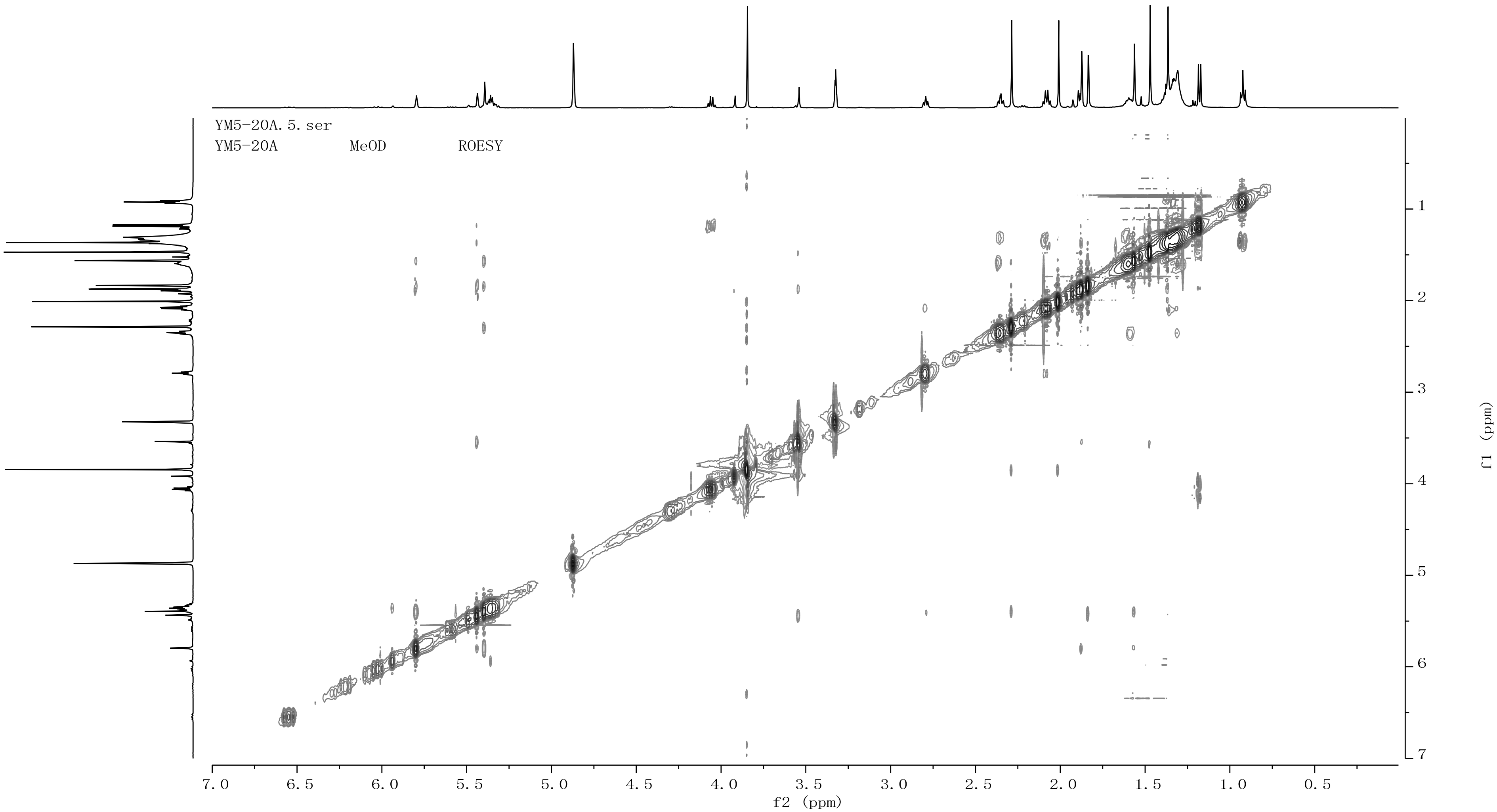

Figure S40. NOESY spectrum of penicicellarusin G (**9**) in CD_3OD
